# Nonwoven-based gelatin/polycaprolactone membrane loaded with ERK inhibitor U0126 for treatment of tendon defects

**DOI:** 10.1186/s13287-021-02679-x

**Published:** 2022-01-10

**Authors:** Yonghui Hou, Bingyu Zhou, Ming Ni, Min Wang, Lingli Ding, Ying Li, Yamei Liu, Wencai Zhang, Gang Li, Jiali Wang, Liangliang Xu

**Affiliations:** 1 Key Laboratory of Orthopaedics & Traumatology, The Second Affiliated Hospital of Guangzhou University of Chinese Medicine, Guangzhou University of Chinese Medicine, Guangzhou, People’s Republic of China; 2 Lingnan Medical Research Center, The First Affiliated Hospital of Guangzhou University of Chinese Medicine, Guangzhou University of Chinese Medicine, Guangzhou, People’s Republic of China; 3 Department of Orthopedics, the First Medical Center, the Fourth Medical Center, Chinese People’s Liberation Army (PLA) General Hospital, Beijing, People’s Republic of China; 4 Departments of Diagnostics of Traditional Chinese Medicine, Guangzhou University of Traditional Chinese Medicine, Guangzhou, People’s Republic of China; 5 Neo Modulus (Suzhou) Medical Sci-Tech Co., Ltd., Suzhou, People’s Republic of China; 6 Department of Orthopaedics & Traumatology, Faculty of Medicine, The Chinese University of Hong Kong, Prince of Wales Hospital, Shatin, Hong Kong People’s Republic of China; 7 Stem Cells and Regenerative Medicine Laboratory, Lui Che Woo Institute of Innovative Medicine, Li Ka Shing Institute of Health Sciences, The Chinese University of Hong Kong, Prince of Wales Hospital, Room 904, 9/F, Shatin, Hong Kong, SAR People’s Republic of China; 8 Biomedical Engineering School, Sun Yat-Sen University, Guangzhou, People’s Republic of China

**Keywords:** Tendon, U0126, GDF6, TSPCs, Nonwoven-based gelatin, Polycaprolactone membrane

## Abstract

**Background:**

Tendon is a major component of musculoskeletal system connecting the muscles to the bone. Tendon injuries are very common orthopedics problems leading to impeded motion. Up to now, there still lacks effective treatments for tendon diseases.

**Methods:**

Tendon stem/progenitor cells (TSPCs) were isolated from the patellar tendons of SD rats. The expression levels of genes were evaluated by quantitative RT-PCR. Immunohistochemistry staining was performed to confirm the presence of tendon markers in tendon tissues. Bioinformatics analysis of data acquired by RNA-seq was used to find out the differentially expressed genes. Rat patellar tendon injury model was used to evaluate the effect of U0126 on tendon injury healing. Biomechanical testing was applied to evaluate the mechanical properties of newly formed tendon tissues.

**Results:**

In this study, we have shown that ERK inhibitor U0126 rather PD98059 could effectively increase the expression of tendon-related genes and promote the tenogenesis of TSPCs in vitro. To explore the underlying mechanisms, RNA sequencing was performed to identify the molecular difference between U0126-treated and control TSPCs. The result showed that GDF6 was significantly increased by U0126, which is an important factor of the TGFβ superfamily regulating tendon development and tenogenesis. In addition, NBM (nonwoven-based gelatin/polycaprolactone membrane) which mimics the native microenvironment of the tendon tissue was used as an acellular scaffold to carry U0126. The results demonstrated that when NBM was used in combination with U0126, tendon healing was significantly promoted with better histological staining outcomes and mechanical properties.

**Conclusion:**

Taken together, we have found U0126 promoted tenogenesis in TSPCs through activating GDF6, and NBM loaded with U0126 significantly promoted tendon defect healing, which provides a new treatment for tendon injury.

**Supplementary Information:**

The online version contains supplementary material available at 10.1186/s13287-021-02679-x.

## Introduction

Tendon is a major component of the musculoskeletal system which connects the muscles to the bone. Injuries to tendon including tendinopathy and tendon rupture are very common orthopedics problems. Tendon disease is one of the most common diagnoses of people engaged in sports professions, accounting for 30% of the total number of injuries diagnosed [1]. Although tendon has selfheal ability, it usually forms scar tissue with lowered biochemical and mechanical properties as compared with intact tendon, leaving the patients prone to re-rupture or impeded motion [2, 3]. Up to now, there still lacks effective treatments for tendon diseases. It is still a great challenge for the formation of functional engineered tendon tissues. Therefore, it is extremely necessary to find better solutions for treating tendon injuries.

The arising of tissue engineering shows promising prospects for tendon tissue regeneration. Many studies have shown that mesenchymal stem cells (MSCs) or tendon stem/progenitor cells (TSPCs) have been successfully applied in tissue engineering to treat tendon injuries [4–6]. MSCs or TSPCs exert their biological functions mainly through paracrine mechanisms or directly differentiate into specific cells. Several factors have been identified to control the expression of tenocyte genes and direct tenogenic differentiation of MSCs or TDSCs, such as transforming growth factor-*β* (*TGFβ*) family of growth factors, *GDF6/7, Scleraxis (Scx), Egr1, Mkx,* etc. [7–10]. However, the mechanisms directing the formation of mature tendon is rarely known. In the search for mechanisms involved in tendon regeneration, extracellular-signal-regulated kinases ERK1/2 signaling has been found as the main checkpoint that regulates the proteolytic breakdown of the tendon matrix [11]. The inhibition of ERK2 with small interfering RNAs is effective in preventing tendon adhesion formation which is one of the most concerning complications after surgical repair of tendon injury [12]. In our previous study, we have found that CFTR mutation would impair tenogenic differentiation and tendon repair by abnormally activating the* β*-catenin/ERK signaling pathway, which could be partially rescued by U0126 [13].

As we know, Type I collagen is the main functional component of the tendon. The other components are a small amount of proteoglycans, glycoproteins and minor collagens [14, 15]. The scaffolds of micro- or nano-fiber have been extensively investigated to regenerate new functional tendon tissue [16, 17]. Poly-*ε*-caprolactone (PCL) scaffolds fabricated by electrospinning have been widely used in soft tissue regeneration as well as tendon engineering. PCL is a low-cost aliphatic linear polyester characterized by its good biocompatibility and bioresorbablity. It has been approved by the U.S. FDA for clinical use [18]. Gelatin is a derivative of collagen which could also be used as a biocompatible scaffold for repairing a wide range of organs or tissues [19–21]. Compared with other synthetic polymers, it better mimics the native microenvironment of tendon tissue. In addition, recent studies have shown that gelatin sponges could be used to control the release of bioactive factors and improve tendon-to-bone healing [22]. And it could also be used to deliver adenovirus to enhance transfection efficiency and tendon healing [23]. Using gelatin and PCL as natural and artificial constituents, the nonwoven-based gelatin/polycaprolactone membrane (NBM) has shown suitability in a preclinical assessment for the treatment of soft tissue defects [24]. In the present study, we investigated the effects of U0126, a specific inhibitor of ERK signal, on tenogenic differentiation of TDSCs and the underlying mechanism, as well as the application of NBM loaded with U0126 on tendon injury healing.

## Materials and methods

### Nonwoven-based membrane (NBM) fabrication

The NBM was generated according to the previously published protocol by Neo Modulus (Suzhou) Medical Sci-Tech Co., Ltd. [24]. The surface topography of NBM was captured by scanning electron microscopy (SEM) by a scanning electron microscope (Zeiss).

### Isolation and culture of TSPCs

The animal experiments were approved by the Animal Research Ethics Committee of Guangzhou University of Chinese Medicine (No. TCMF1-2019053). 10-week-old male SD rats or GFP-transgenic SD rats were used in this study. The details of TSPCs isolation and culture have been described previously [25]. Briefly, the patellar tendons were excised and minced, digested with type I collagenase (2 mg/ml; Sangon). The released cells were washed with PBS and resuspended in low glucose DMEM supplemented with 10% FBS and 2 mM L–glutamine. The surface markers of TSPCs were characterized by flow cytometry. The TSPCs used in this study were between passages 3 and 8, and in each section of the experiment, the cells were in the same passage.

### Cell viability assay

The cells (3 × 10^3^ per well) were subcultured in a 96-well plate. After 24 h of incubation, the medium was changed into U0126 containing medium at different concentrations. Cells were incubated at 37 °C for 1 and 3 days. The cell proliferation was determined using methyl thiazolyl tetrazolium (MTT) reduction assay. After incubation, cells were treated with the MTT solution (final concentration, 0.5 mg/ml) for 4 h at 37 °C. The dark blue formazan crystals formed in intact cells were solubilized with 100 μL DMSO and the plate was shaken for 10 min. The absorbance at 570 nm was measured with a microplate reader.

### Tenogenic differentiation of TSPCs

The TSPCs were plated in 6-well plates (2 × 10^5^ cell/well) and cultured until the cells reached confluence. Then the medium was changed to the tenogenic induction medium with ascorbic acid (25 μM) and CTGF (25 ng/ml) at 37 °C, 5% CO_2_. The medium was changed every 2 or 3 days. Two weeks later, the expressions of tendon-related markers were evaluated by quantitative real-time RT-PCR (qRT-PCR). At 2 weeks after tenogenic induction, the cells were stained with Sirius Red (0.1%). For quantification, Sirius Red was eluted with 0.1 N sodium hydroxide, and the optical density at 540 nm was determined using a spectrophotometer.

### RNA extraction and quantitative real-time PCR

Total RNA was extracted using Takara Mini BEST Universal RNA Extraction Kit according to the manufacturer’s instructions. Briefly, the cells were lysed with Buffer RL reagent for 10 min. DNase I was used to remove contaminating DNA in total RNA. The first-strand cDNA was synthesized using Prime Script RT Master Mix (Perfect Real Time). Real-time PCR was performed using the CFX96 Real-Time PCR Detection System (Bio-Rad, USA). Amplification conditions were as follows: first at 95 °C for 5 min, and then 45 cycles of 95 °C for 15 s and 60 °C for 60 s. Primer sequences were listed in Additional file 2: Table S1. The relative quantification of gene expression was normalized to the expression level of *GAPDH*. And the expression levels of genes in the control group were arbitrarily set to 1.

### RNA-seq and data analysis

Total RNA was obtained from the TSPCs treated with or without U0126 using TRIzol Reagent (Takara, Dalian, China). The quality and integrity of total RNA samples were assessed using a 2100 Bioanalyzer or a 2200 TapeStation (Agilent Technologies) according to the manufacturer’s instructions. The preparation of whole transcriptome libraries and deep sequencing were performed by the Sangon Biotech Corporation (Shanghai, China). The differential genes (log2ratio ≥ 1 or ≤ − 1) identified by RNA-seq have been uploaded in Additional file 3: Table S2. DAVID bioinformatics tool was also used for functional annotation enrichment and clustering.

### In vivo neo-tendon formation by engineered scaffold-free tendon tissue in nude mice

In order to demonstrate the formation of neo-tendon tissue in vivo, the cell sheets formed by TSPCs treated with or without U0126 were transplanted to the dorsal sites of nude mice. Briefly, a total of 8 mice were used; after anesthetized with ketamine and xylazine, an incision was made on the dorsum and a subcutaneous pocket was created to expose the posterior midline. The cell sheet was sutured to the posterior midline at both ends, there was tensile strength on the tendon graft with the mice movement. Six weeks later, the tissues were harvested and subjected to histological analysis.

### Patellar tendon injury and repair animal model

Forty SD male rats (10 weeks old, about 300 g) were used in this study. A 2.0 mm wide tendon defect was created from the distal apex of the patella to the insertion of the tibia tuberosity with two stacked sharp blades according to our well-established protocol. U0126 was dissolved in DMSO and diluted with PBS. Then NBM scaffold was immersed in the U0126 solution overnight at 4 °C. The operated rats were divided into four groups: (a) Blank control group; (b) NBM-PBS group; (c) NBM-U0126 (100 mΜ) group; and (d) NBM-U0126 (200 mΜ) group. The NBM loaded with PBS and/or U0126 was placed in the tendon defect and sutured to the patellar bone and tibia tuberosity using Ethicon 6-0 suture. At 4 weeks after surgery, the animals were sacrificed by an overdose of ketamine and xylazine, and the patellar tendons were harvested for histological examination and biomechanical test.

### Biomechanical testing

The procedure of biomechanical testing was described in a previous study [26]. Generally, the patellar tendon-tibia composite was isolated. Then the regenerated tissue in the window wound was isolated by excising the healthy tendon. The composite was fixed on a custom-made testing jig with two clamps. The lower one was used to fix the tibia shaft and plateau while the upper one was used to fix the proximal patella, the quadriceps muscles, and its tendons without creating mechanical stress to the junction and the mid-substance. Hounsfield H25KS mechanical testing machine (Tinius Olsen Ltd, Salfords, UK) was used for biomechanical testing. The test was performed at a speed of 40 mm/min, using a 50 N loading cell. The ultimate stress (N/mm^2^) was calculated based on the ultimate load divided by the cross-sectional area at the breaking point measured by a high-resolution Vevo 770 animal ultrasound system (Visualsonics, Toronto) with images taken immediately prior to the biomechanical test. The Young’s modulus (N/mm^2^) was calculated from the linear slope of a stress strain curve.

### Histology and immunohistochemistry

The regenerated patellar tendon tissues were washed with PBS, fixed in buffered formalin, embedded in paraffin and sectioned for histological examination. Immunohistochemistry was done as described previously [26]. Briefly, after deparaffination, the sections were rehydrated, quenched of endogenous peroxidase activity and subject to antigen retrieval. After blocking with 5% normal donkey and goat serum, the sections were incubated with specific antibodies against Tnmd (sc-98875, Santa Cruz Biotechnology), Collagen I (AF7001, Affbiotech), GDF6 (bs-11843R, Bioss), Egr1 (bs-1076R, Bioss), OPN (bs-0026R, Bioss), OCN (bs-0470R, Bioss), MMP13(bs-0575R, Bioss) and Scx (sc-87425, Santa Cruz Biotechnology) at dilution of 1:200 at 4 °C overnight. Goat anti-rabbit horseradish peroxidase (HRP)-conjugated secondary antibody and donkey anti-goat horseradish peroxidase (HRP)-conjugated secondary antibody (Santa Cruz Biotechnology; dilution 1:200) were then added for 30 min, respectively. Afterward, the sections were rinsed, counterstained in hematoxylin or methylgreen, dehydrated with graded ethanol and xylene, and mounted with p-xylene-bis-pyridinium bromide (DPX) permount (Sigma Aldrich, St Louis, MO, USA). All incubation times and conditions were strictly controlled. The sections were examined under light microscopy (DMRXA2, Leica, Germany).

### Data analysis

All data were presented as mean ± SD. The statistical analysis was performed using one-way analysis of variance (one-way ANOVA). A value of *p* < 0.05 was considered statistically significant. At least three sets of independent experiments were performed for each assay.

## Results

### U0126 promoted tenogenesis in rat TSPCs

**Fig. 1:**
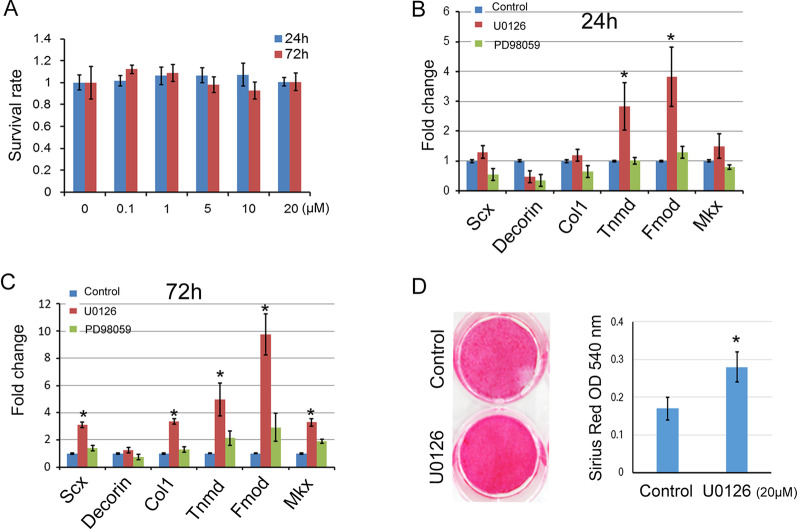
U0126 promoted the tenogenesis of TSPCs. **A** Viability of rat TSPCs treated with U0126 at different concentrations. The cells were incubated with U0126 (0.1 μM to 20 μM) for 24 h and 72 h, then MTT assay was performed to test the cell viability. (B&C) Total RNA was extracted from TSPCs treated with or without U0126 (20 μM) or PD98059 (20 μM) for 24 h (**B**) and 72 h (**C**). The relative expression levels of *Scx, Decorin, Collagen type I, Tnmd, Fmod* and *Mkx* were evaluated by qRT-PCR. GAPDH was used as an internal control. The data was expressed as mean ± SD (*n* = 3), *p < 0.05. **D** The TSPCs were treated with tenogenic induction medium supplemented with or without U0126 (20 μM) for 14 days, then Sirius Red staining was performed. And the Sirius Red was quantified using a spectrophotometer at 540 nm

In order to evaluate the effect of U0126 on the tenogenesis of rat TSPCs, the cells were treated with U0126 at different concentrations for 24 h and 72 h. The MTT assay showed that U0126 (0.1 μM to 20 μM) did not affect the cell viability of TSPCs (Fig. 1A). So we chose 20 μM U0126 for further study in vitro. The specific effect of U0126 on tenogenesis of rat TSPCs was investigated at 24 h and 72 h after the treatment, compared with another ERK inhibitor-PD98059 (Fig. 1B, C). We found that U0126 could significantly increase the expression levels of tenogenesis-related genes such as *Scx, Col1, Tnmd, Fmod and Mkx*. While, PD98059 did not show an obvious promoting effect. The Sirius Red staining at 2 weeks after tenogenic induction also demonstrated that U0126 significantly promoted the tenogenesis of TSPCs (Fig. 1D).

### Ectopic tendon formation of cell sheet in nude mice

**Fig. 2:**
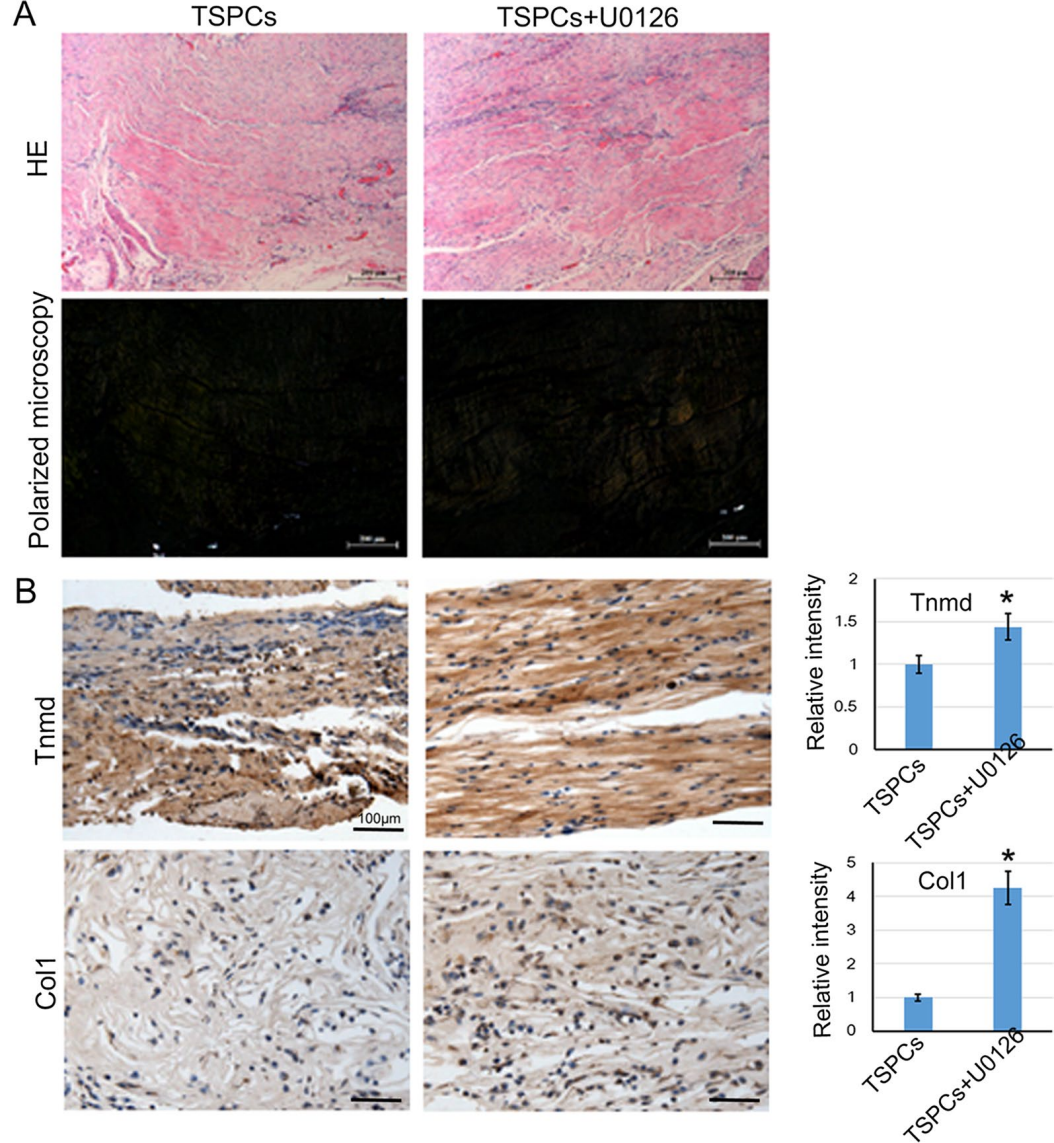
U0126 promoted ectopic tendon formation in nude mice. The cell sheets formed by TSPCs with U0126-treatment or not were implanted into the nude mice. After 6 weeks of transplantation, the samples were collected, and sectioned. **A** HE staining of the sections, and visualized under polarized microscopy. Scale bar = 200 μm. **B** Immunohistochemistry (IHC) staining of sections. Anti-Tnmd and Col1a1 antibodies were used for IHC staining to observe their expression in the newly formed tendon-like tissue. The relative intensity was analyzed by Image J software (IHC Toolbox). The data was expressed as mean ± SD (*n* = 3), *p < 0.05. Scale bar = 100 μm

To observe the formation of neo-tendon tissue in vivo, the cell sheets formed by U0126-treated or not treated TSPCs were implanted into the nude mice. After 6 weeks of transplantation, there were loosely deposited collagens in the tendon-like structure as depicted by HE staining (Fig. 2A). Immunohistochemistry staining of Tnmd and collagen type I (Col I) was also performed to observe their expression in the newly formed tendon-like tissue. And the result showed that the expression of Tnmd and Col I in the U0126 treated group was higher than that of the control group (Fig. 2B). We also observed that the alignment of cells in the newly formed tendon-like tissue was along with the collagen fibers which resembled more like the intact tendon; while the control group showed a more randomly aligning pattern.

### RNAseq analysis of the gene expression profiles of TSPCs treated with or without U0126

**Fig. 3:**
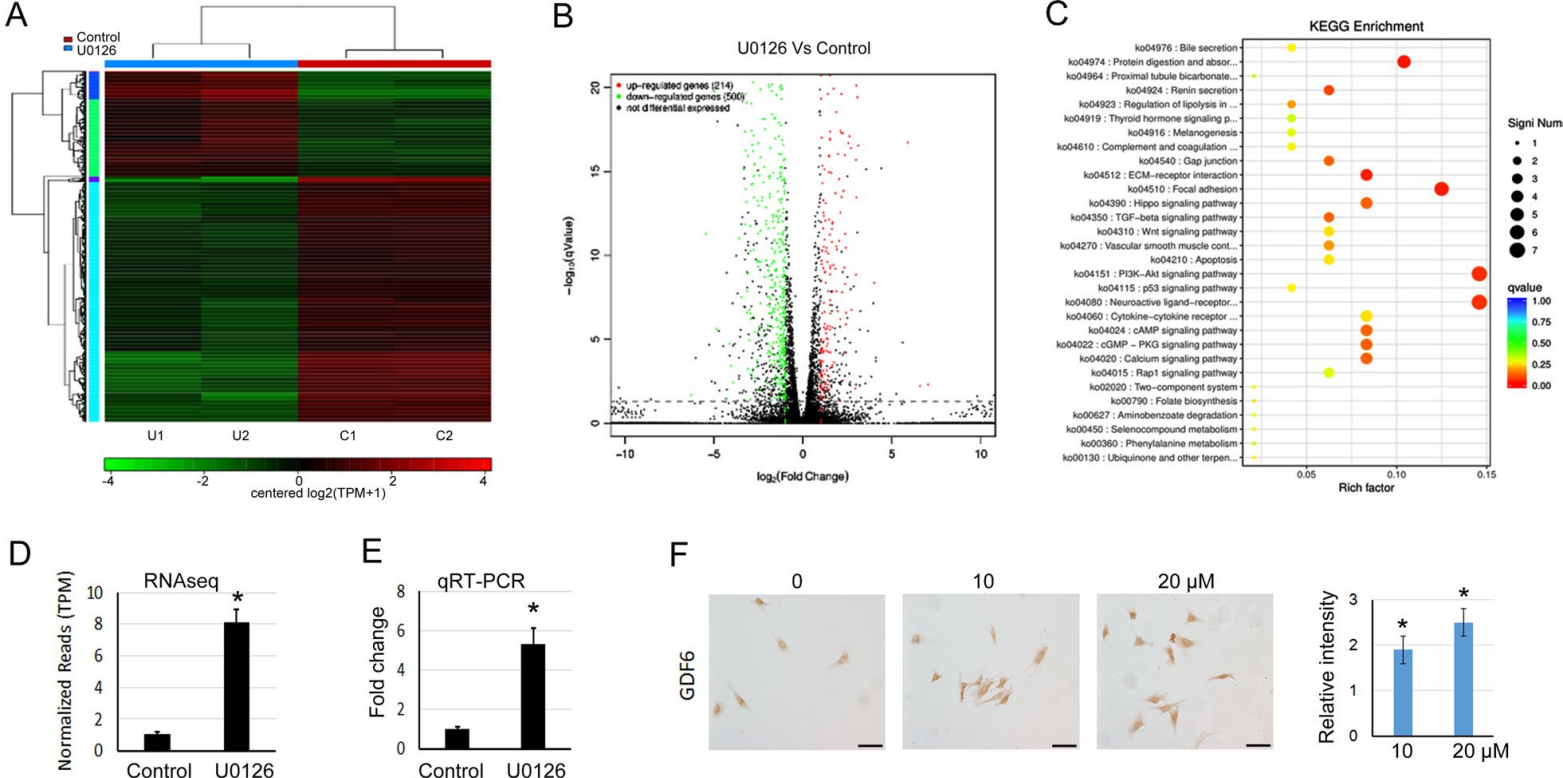
RNAseq analysis of gene expression profiles of TSPCs treated with or without U0126. **A** Heatmap depicting expression levels of genes between U0126-treated or control TSPCs. In total, 716 genes were differentially expressed between these two groups of TSPCs. **B** Volcano map of the differentially expressed genes in U0126-treated or control TSPCs. **C** The TOP 30 enriched KEGG pathways in U0126-treated TSPCs. **D** RNAseq analysis showed *GDF6* was significantly increased in U0126-treated TSPCs, as compared with control TSPCs. **E** The relative expression level of *GDF6* was evaluated by qRT-PCR in U0126-treated TSPCs. GAPDH was used as an internal control. The data was expressed as mean ± SD (*n* = 3), *p < 0.05. **F** Immunocytochemical staining of GDF6 in TSPCs treated with different concentrations of U0126. And the relative intensity was quantified with Image J software. The data was expressed as mean ± SD (*n* = 3), *p < 0.05. Scale bar = 100 μm

To analyze the underlying mechanism related to the promoting effect of U0126 on tenogenic differentiation of TSPCs, RNAseq was performed to check the gene expression profiles of TSPCs treated with U0126 or not, respectively. The heatmap and volcano map were shown in Fig. 3A, B. 215 up-regulated and 501 down-regulated genes with log_2_ratio above 1 were discovered in U0126 treated TSPCs compared with control TSPCs. The KEGG (Kyoto Encyclopedia of Genes and Genomes) analysis revealed that many signaling pathways were enriched, the Top 30 of which were shown in Fig. 3C. The focal adhesion, extracellular matrix (ECM)-receptor interaction and TGF-beta signaling pathways stood out from the KEGG analysis, which have been considered as important factors involved in tenogenesis, tendon development and healing. Among all the genes identified, we found that *GDF6*, an important member of TGF-*β* signaling which has been well known as an indispensable factor for tenogenesis and tendon development, was significantly increased by U0126 which was confirmed by qRT-PCR and immunocytochemical analysis (Fig. 3D–F).

### NBM loaded with U0126 promoted tendon defect healing

**Fig. 4:**
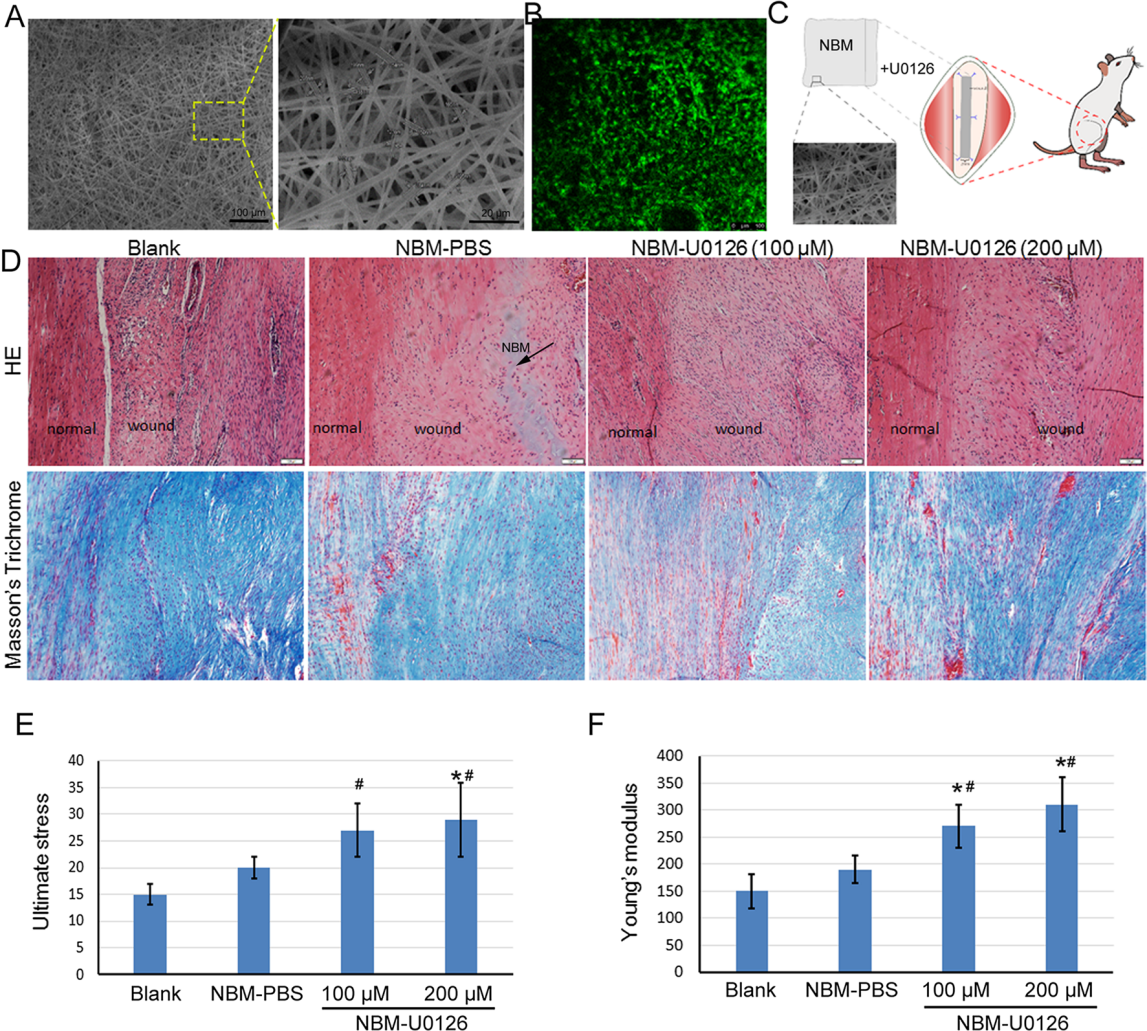
NBM loaded with U0126 promoted tendon defect healing. **A** SEM documentation of NBM. **B** Fluorescence microscopy of TSPCs on NBM. The GFP-labeled TSPCs were cultured on the NBM for 7 days, then the cells were visualized by confocal fluorescence microscopy. **C** Diagram illustrating the implantation of NBM loaded with U0126 into the patellar tendon defects. **D**–**F** At 4 weeks after the operation, the tendon samples were collected for histological staining and biomechanical testing. HE and Masson’s trichrome staining of tendon injury in a rat model at 4 weeks after surgery (**D**). Scale bar = 100 μm. (**E**, **F**) The mechanical properties of the injured tendon tissues were analyzed by biomechanical testing. The data was expressed as mean ± SD (*n* = 5), *p < 0.05, compared with blank control; ^#^*p* < 0.05, compared with NBM group

Based on the previously described intrinsic features of NBM, we hypothesized it would have well cellular biocompatibility. We used high-resolution SEM to visualize the fibers of NBM, it showed that the NBM displayed a random but regular configuration (Fig. 4A). When TSPCs (labeled with GFP) were cultured on it, the cells displayed normal cell morphology (Fig. 4B).

In order to evaluate the effect of NBM and U0126 on tendon injury healing, different dosages of U0126 were loaded into NBM. The release kinetics of U0126 from NBM in vitro was shown in Additional file 1: Fig. S1. Then the rat patellar tendon injury model was established, and the NBMs loaded with U0126 or PBS were transplanted into the defect region (Fig. 4C). At 4 weeks after the operation, the tendon samples were collected for histological analysis and biomechanical testing. Both HE and Masson’s trichrome staining indicated that NBM loaded with U0126 group had more matrix and collagen formation in the wound region as compared with the blank and NBM + PBS group (Fig. 4D). In the blank control group, there was still a gap between normal tendon tissue and the wound area; the wound area was filled with inflammatory cells and blood vessels, and the cells were randomly oriented with fewer collagen fibers. For the NBM-PBS group, we could still observe the un-degraded NBM and the randomly orientated cells. While, in the NBM loaded with U0126 groups, we found better collagen fiber and cell alignments which more closely resembled normal tendon tissues than that of control groups. The biomechanical testing result also showed that the ultimate stress and Young’s modulus were significantly higher NBM-U0126 group compared to that of control groups (Fig. 4E, F).

**Fig. 5:**
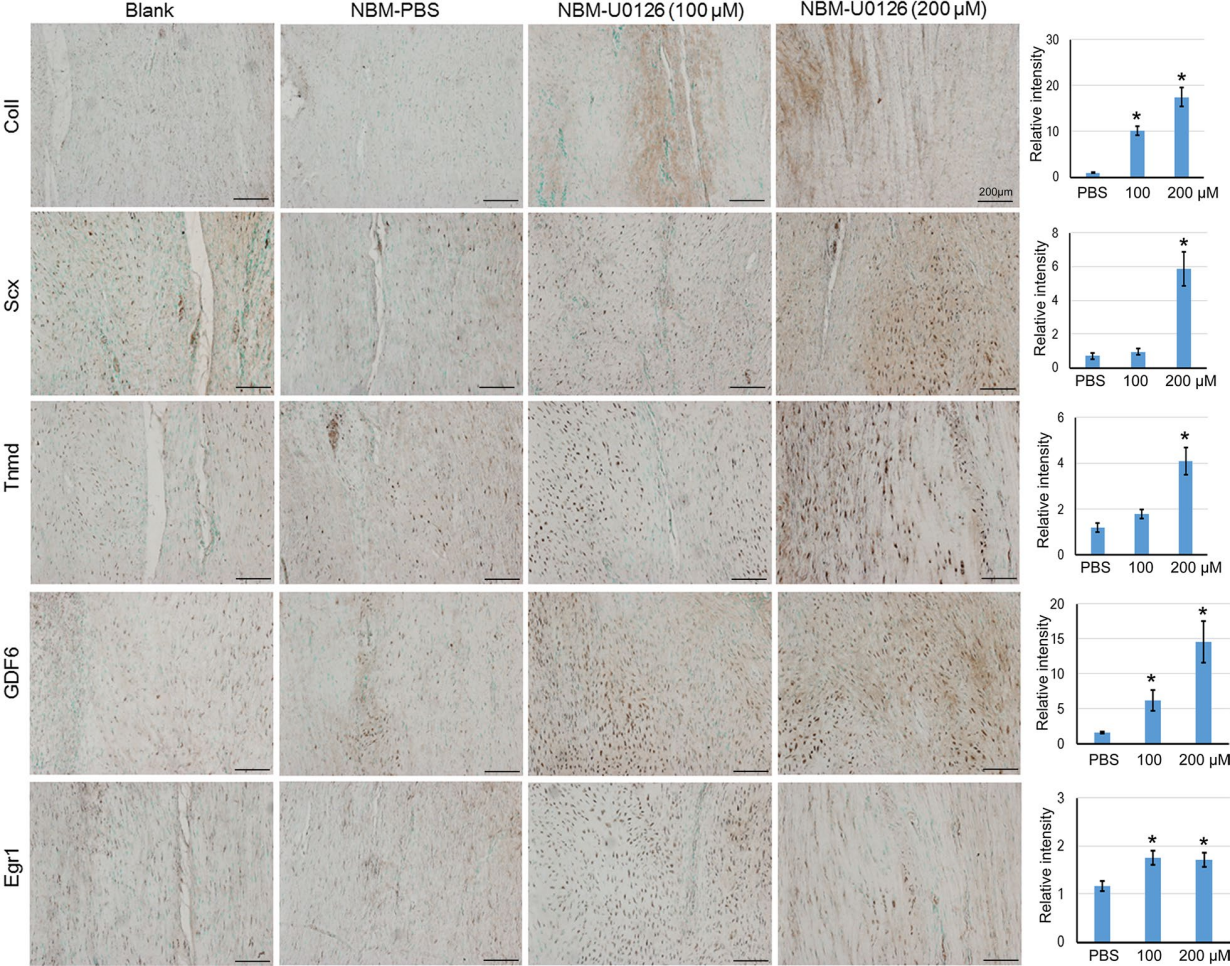
U0126 increased tendon-related makers in injured tendon tissues. 4 weeks after the operation, the tendon tissues were collected, sectioned for IHC staining with anti-Egr1, Scx, Col1a1, Tnmd and GDF6 antibodies, respectively. The relative intensity was analyzed by Image J software (IHC Toolbox). The data was expressed as mean ± SD (*n* = 3), *p < 0.05. Scale bar = 200 μm

**Fig. 6:**
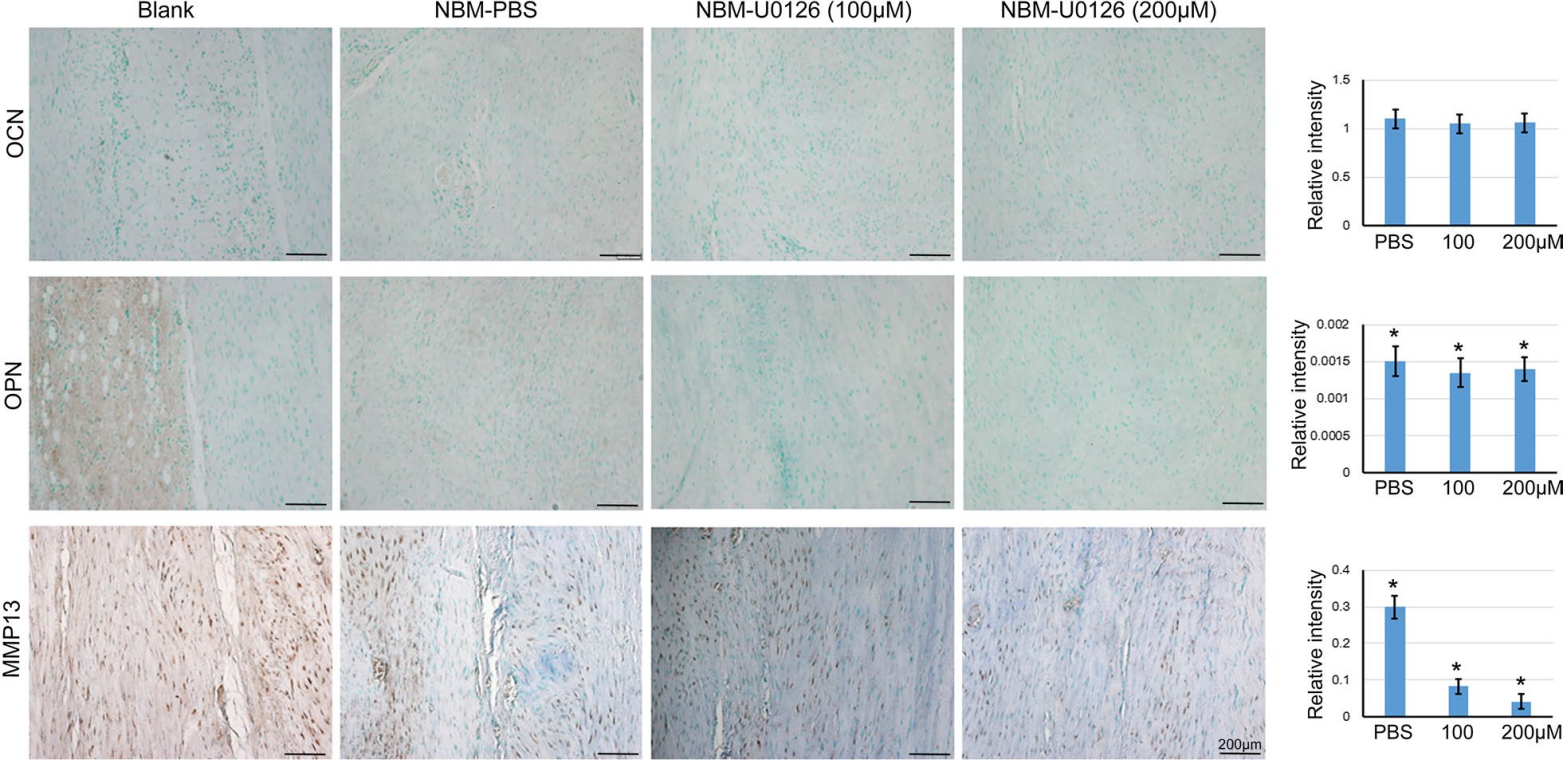
NBM loaded with U0126 inhibited ossification and inflammation in injured tendon tissues. 4 weeks after the operation, the tendon tissues were collected, sectioned for IHC staining with anti-OCN, OPN and MMP13 antibodies, respectively. Scale bar = 200 μm

In addition, we also observed the increased expression of markers related to tenogenic differentiation and tendon development, including Egr1, Scx, Col1a1, and Tnmd, as well as GDF6 in NBM-U0126 groups versus groups at 4 weeks after surgery (Fig. 5). In particular, the higher U0126 group showed better outcomes than the lower group. As tendon calcification and inflammation are common problems in tendon injury, so we also checked the expression of OCN, OPN and MMP13 in each group (Fig. 6). We could clearly observe that in the blank control group, there was apparent OPN expression, while no positive signal was seen in the other three groups, implying that the cells in the wound area were correctly directed by NBM to avoid wrong differentiation. The expression level of MMP13 in each group was much lower than that of the blank control group, and the higher dosage of U0126 the better outcome. Taken together, our result showed that NBM had well cellular biocompatibility allowing tendon cells to grow in both in vitro and in vivo. Most importantly, the healing of tendon defects was significantly promoted by NBM in combination with U0126 in a rat model of tendon injury.

## Discussion

The formation of scar tissue after tendon injury greatly lowered the biochemical and mechanical properties of tendon, leaving the patients prone to re-rupture or impeded motion [27]. Tissue engineering is a promising solution for achieving functional tendon healing [28]. In the present study, we have shown that U0126 promotes the tenogenesis of TSPCs by activating *GDF6* which is an important factor of the TGFβ superfamily regulating tendon development and tenogenesis. In addition, NBM has shown well cellular biocompatibility allowing tendon cells to grow in both in vitro and in vivo. Most importantly, when it is used in combination with U0126, the healing of tendon defects is significantly promoted in a rat model of tendon injury.

U0126 has been used as a specific inhibitor of the ERK1/2 signaling pathway by selectively inhibiting MEK-1 and MEK-2 [29]. It has been found that ERK1/2 activity is required for tendon deterioration, ERK1/2 is highly phosphorylated in degrading tendon fascicles [11]. And down-regulating ERK1/2 phosphorylation has been shown to ameliorate tendon adhesion and joint adhesion [30, 31]. Our findings showed that U0126 could promote tenogenesis of TSPCs, while the other ERK1/2 inhibitor PD98059 had no such effect, meaning that this effect might be attributed to the novel functions of U0126 apart from its role as ERK1/2 inhibitor. Then we used RNAseq analysis to implore the underlying mechanisms of how U0126 promoted tenogenesis. The KEGG analysis revealed that many signaling pathways were enriched, among which we found the focal adhesion, extracellular matrix (ECM)-receptor interaction, PI3K-Akt and TGF-beta signaling pathways were activated by U0126. These pathways, especially the TGF-beta signaling pathway, have been considered as important factors governing tenogenesis, tendon development, as well as tendon injury healing [32–35]. GDF6 stood out in our further analysis as it was an important member of TGF-*β* signaling which was significantly increased by U0126. *GDF6* has well known as an indispensable factor for tenogenesis and tendon development. In 1997, GDF5, 6 and 7 have been demonstrated to induce neotendon/ligament formation when implanted at ectopic sites in vivo [36]. And the role of *GDF6* in tendon matrix modeling was first reported by Borjana Mikic et al. in 2009 [37]. They have demonstrated that a null mutation in *GDF6* is associated with substantially lower levels of tail tendon collagen content (− 33%) in four-week-old male mice, which has direct functional consequences for the mechanical integrity of the tissue (45–50% reduction in material properties) [37]. Our previous study also showed *GDF6* has promoting effect on the tenogenic differentiation of BMSCs [38].

The NBM, composed of electrospun gelatin and polycaprolactone nanofiber nonwovens, mimics the ECM of tissues. It was fabricated by electrospinning of in situ cross-linked GE nanofiber nonwovens and subsequent lamination with electrospun PCL nanofiber nonwovens by heating the layer assemblies above the melting temperature of PCL. It has been used to treat soft tissue defects [24]. The present study showed that NBM had good biocompatibility and bioresorbablity. Guang Yang et al. have developed a novel composite scaffold fabricated by co-electrospinning of PCL and methacrylated gelatin (mGLT), and the human adipose-derived stem cells could impregnate in the scaffold to form tendon-like features [39]. But unfortunately, they did not perform in vivo study to prove its effectiveness for tendon tissue engineering. Recently, it has been reported that the biomimetic PCL/gelatin-aligned scaffolds have been used for the mechanical restoration of the injured tendon in a rabbit model [40]. It should be noted that different topographic cues of scaffolds may cause different performance in mechanics and cell differentiation capacities [41, 42]. The NBM we used in this study displayed a random but regular configuration, it is easy to be degraded in vivo after 4 weeks when the cells like tenocytes or TSPCs impregnate in the scaffold. To some extent, it would be better if the aligned scaffolds were used. On the other hand, if chemicals instead of stem cells are loaded in the scaffolds, the topographic cues of scaffolds may not have a huge impact on tendon regeneration in vivo.

## Conclusion

In this study, we have demonstrated that U0126 could promote tenogenesis of TSPCs by activating *GDF6* expression. Most importantly, we also developed a novel combination of NBM and U0126 which could be used to promote tendon injury healing in a rat tendon defect model.

## Supplementary Information

**Additional file 1**. **Fig. S1**. Release kinetics of U0126 from NBM scaffold in vitro.

**Additional file 2**. **Table S1**. Sequences of primers for real-time PCR.

**Additional file 3**. **Table S2**. Differentially expressed genes (log2ratio ≥ 1 or ≤ -1) in U0126-treated TSPCs.

## Data Availability

The dataset supporting the conclusions of this article is available in the NCBI Sequence Read Archive (SRA) repository under the submission ID PRJNA786244, https://dataview.ncbi.nlm.nih.gov/object/PRJNA786244?reviewer=hj8mnkj2siqp86541a9t0q3ig3. The other data that support the findings of this study are available on request from the corresponding author.

## Abbreviation

TSPCs: Tendon stem/progenitor cells
MSCs: Mesenchymal stem cells
PBS: Phosphate-buffered saline
NBM: Nonwoven-based gelatin/polycaprolactone membrane
OCN: Osteocalcin
TGFβ: Transforming growth factor-*β*
Scx: Scleraxis
OPN: Osteopontin
PCL: Poly-*ε*-caprolactone
KEGG: Kyoto encyclopedia of genes and genomes
ECM: Extracellular matrix
qRT-PCR: Quantitative real-time RT-PCR
