# Supplementary figures and images for: Nonwoven-based gelatin/polycaprolactone membrane loaded with ERK inhibitor U0126 for treatment of tendon defects
Source: Stem Cell Res Ther. 2022 Jan 10;13:5. doi: 10.1186/s13287-021-02679-x (PMC8744263; doi:10.1186/s13287-021-02679-x)

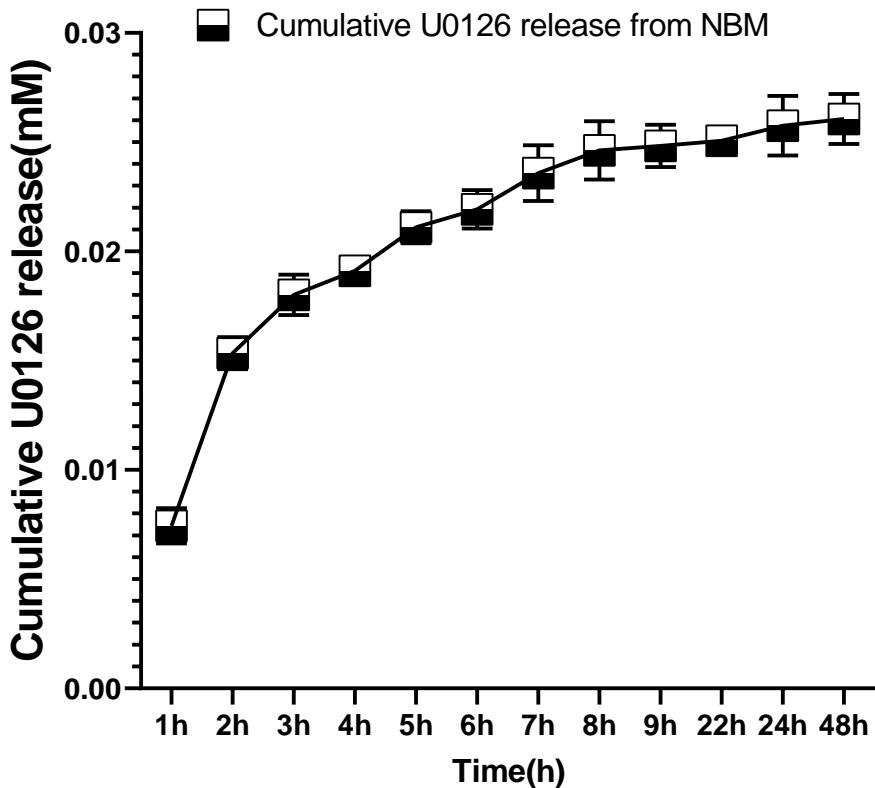

Supplement: Supplementary file 1 — Additional file 1. Fig. S1. Release kinetics of U0126 from NBM scaffold in vitro. [file 13287_2021_2679_MOESM1_ESM.pdf]
