# Supplementary material for: Nonwoven-based gelatin/polycaprolactone membrane loaded with ERK inhibitor U0126 for treatment of tendon defects
Source: Stem Cell Res Ther. 2022 Jan 10;13:5. doi: 10.1186/s13287-021-02679-x (PMC8744263; doi:10.1186/s13287-021-02679-x)
Supplement: Supplementary file 2 — Additional file 2. Table S1. Sequences of primers for real-time PCR. [file 13287_2021_2679_MOESM2_ESM.docx]

**Supplementary Table 1.** Sequences of primers for real time PCR.

| *Gene Name* | *Forward primer sequence (5’ to 3’)* | *Reverse primer sequence (5’ to 3’)* | |
| --- | --- | --- | --- |
| *Scx* | CTGGCCTCCAGCTACATCTC | CGGTCCTTGCTCAACTTTCT |  |
| *Tnmd* | TGCTGTAGAAAGTGTGCTCCA | GATTTGTGGACTGGTGTTTGG |  |
| *Decorin* | ACAAGTTTCCTGGGCTGGAC | AGGCCCCCTCTTTGATCTCT |  |
| *GAPDH* | CGTAAAGACCTCTATGCCAACA | CGGACTCATCGTACTCCTGCT |  |
| *FMOD*  *Col1*  *GDF6*  *Mkx* | AGAAGTTCACGACGTCCACC CACTGGTGATGCTGGTCCTG  TGCACGTGAACTTCAAGGAG  CCATTCTTTCGCCTTGCTCC | CAGCCTCCTTGAGCTAGACC  CGAGGTCACGGTCACGAAC  CCCGCGTCGATGTATAGAAT  TGCAGAGGTAGAAATGCGGG |  |
