# Supplementary material for: Nonwoven-based gelatin/polycaprolactone membrane loaded with ERK inhibitor U0126 for treatment of tendon defects
Source: Stem Cell Res Ther. 2022 Jan 10;13:5. doi: 10.1186/s13287-021-02679-x (PMC8744263; doi:10.1186/s13287-021-02679-x)
Supplement: Supplementary file 3 — Additional file 3. Table S2. Differentially expressed genes (log2ratio ≥ 1 or ≤ -1) in U0126-treated TSPCs. [file 13287_2021_2679_MOESM3_ESM.docx]

| Gene id | MeanTPM (U0126) | MeanTPM (DMSO) | log2FoldChange | pValue | qValue | result | GeneName |
| --- | --- | --- | --- | --- | --- | --- | --- |
| ENSRNOG00000002789 | 9.843926 | 0.0001 | 16.58695 | 3.56E-09 | 8.67E-08 | up | AABR07039648.1 |
| ENSRNOG00000002331 | 12.75769 | 0.098777 | 7.012976 | 0.000559 | 0.004933 | up | Aldh3a1 |
| ENSRNOG00000045586 | 30.16019 | 0.31748 | 6.569838 | 0.000663 | 0.005723 | up | AABR07073186.1 |
| ENSRNOG00000011841 | 6.157816 | 0.104864 | 5.875827 | 2.89E-19 | 1.97E-17 | up | Map2 |
| ENSRNOG00000050333 | 10.40222 | 0.207509 | 5.64757 | 0.008019 | 0.046351 | up | AABR07037465.1 |
| ENSRNOG00000051993 | 13.57072 | 0.422488 | 5.005443 | 1.01E-32 | 1.71E-30 | up | Gdf10 |
| ENSRNOG00000009892 | 9.064261 | 0.485454 | 4.222783 | 1.17E-80 | 1.51E-77 | up | Adamts15 |
| ENSRNOG00000001652 | 36.23039 | 2.265992 | 3.998986 | 1.45E-10 | 4.18E-09 | up | Erg |
| ENSRNOG00000024899 | 24.57983 | 1.716496 | 3.839937 | 2.23E-26 | 2.42E-24 | up | Cxcl13 |
| ENSRNOG00000010478 | 38.86582 | 2.816052 | 3.786756 | 1.74E-51 | 7.07E-49 | up | LOC500712 |
| ENSRNOG00000045683 | 49.40603 | 3.580429 | 3.786483 | 9.67E-50 | 3.56E-47 | up | LOC102553715 |
| ENSRNOG00000008915 | 26.10077 | 1.946995 | 3.744771 | 1.27E-62 | 9.34E-60 | up | Prima1 |
| ENSRNOG00000002382 | 51.62659 | 4.147309 | 3.637867 | 7.49E-49 | 2.69E-46 | up | LOC102553715 |
| ENSRNOG00000010325 | 38.49406 | 3.191979 | 3.592113 | 1.49E-54 | 7.96E-52 | up | Ptger3 |
| ENSRNOG00000014293 | 11.31553 | 0.963783 | 3.553453 | 6.94E-40 | 1.68E-37 | up | Nkd1 |
| ENSRNOG00000061910 | 355.598 | 30.38199 | 3.548959 | ######## | 1.70E-97 | up | Igfbp3 |
| ENSRNOG00000026953 | 8.831499 | 0.773485 | 3.513212 | 1.68E-32 | 2.83E-30 | up | Gpr88 |
| ENSRNOG00000002548 | 1657.814 | 145.6183 | 3.509018 | ######## | ######## | up | Tnn |
| ENSRNOG00000016717 | 16.67932 | 1.707109 | 3.288433 | 5.83E-27 | 6.58E-25 | up | Gas2 |
| ENSRNOG00000007810 | 19.14556 | 2.110052 | 3.181659 | 1.37E-62 | 9.61E-60 | up | Gdf6 |
| ENSRNOG00000039560 | 19.75453 | 2.323191 | 3.088004 | 4.37E-19 | 2.91E-17 | up | Omd |
| ENSRNOG00000004578 | 21.75714 | 2.588254 | 3.071438 | 5.27E-31 | 7.69E-29 | up | Cthrc1 |
| ENSRNOG00000019926 | 106.2816 | 12.67304 | 3.068057 | 2.97E-51 | 1.18E-48 | up | Ramp1 |
| ENSRNOG00000002365 | 43.26663 | 5.164562 | 3.066537 | 1.24E-33 | 2.13E-31 | up | Itm2a |
| ENSRNOG00000048472 | 9.584646 | 1.168488 | 3.036083 | 4.82E-22 | 3.86E-20 | up | Comp |
| ENSRNOG00000046658 | 13.56873 | 1.686399 | 3.008268 | 6.85E-16 | 3.45E-14 | up | LOC103690116 |
| ENSRNOG00000015902 | 12.74514 | 1.594689 | 2.9986 | 8.82E-23 | 7.66E-21 | up | Cpxm2 |
| ENSRNOG00000026607 | 384.1045 | 48.67449 | 2.980261 | 4.49E-11 | 1.39E-09 | up | Tnfsf18 |
| ENSRNOG00000051399 | 6.675227 | 0.848971 | 2.97503 | 3.32E-18 | 2.07E-16 | up | Col10a1 |
| ENSRNOG00000018570 | 122.856 | 15.81346 | 2.957744 | 7.43E-73 | 7.18E-70 | up | C1qtnf3 |
| ENSRNOG00000030715 | 6.430355 | 0.841043 | 2.934647 | 0.00104 | 0.008503 | up | Cfh |
| ENSRNOG00000019851 | 88.85614 | 12.26551 | 2.856864 | 5.19E-42 | 1.36E-39 | up | Cox6a2 |
| ENSRNOG00000015410 | 192.3929 | 28.59787 | 2.750076 | 6.65E-51 | 2.57E-48 | up | Aspn |
| ENSRNOG00000010545 | 5.660381 | 0.856437 | 2.72448 | 7.76E-09 | 1.79E-07 | up | Mrap2 |
| ENSRNOG00000000885 | 6.868552 | 1.063107 | 2.691719 | 6.22E-19 | 4.09E-17 | up | Auts2 |
| ENSRNOG00000012036 | 10.23113 | 1.626745 | 2.652906 | 2.93E-31 | 4.39E-29 | up | Pcsk5 |
| ENSRNOG00000014948 | 15.28121 | 2.454813 | 2.638073 | 5.12E-27 | 5.86E-25 | up | Osgin1 |
| ENSRNOG00000020622 | 5.189642 | 0.840116 | 2.626975 | 1.08E-19 | 7.61E-18 | up | Cilp2 |
| ENSRNOG00000001854 | 12.12725 | 2.066965 | 2.552667 | 3.01E-19 | 2.03E-17 | up | Tmtc1 |
| ENSRNOG00000029911 | 207.3464 | 35.64711 | 2.540186 | 6.95E-94 | 1.79E-90 | up | Cilp |
| ENSRNOG00000014721 | 20.61341 | 3.63952 | 2.501763 | 3.25E-57 | 1.93E-54 | up | Ahrr |
| ENSRNOG00000001469 | 3080.245 | 547.9629 | 2.490895 | 8.24E-85 | 1.27E-81 | up | Eln |
| ENSRNOG00000050431 | 146.8145 | 27.28731 | 2.427692 | 4.40E-06 | 6.21E-05 | up | Aspnl1 |
| ENSRNOG00000011502 | 152.4615 | 28.49317 | 2.419757 | 1.21E-48 | 4.25E-46 | up | Col9a2 |
| ENSRNOG00000029792 | 225.1043 | 42.29614 | 2.411996 | 1.16E-08 | 2.58E-07 | up | NEWGENE_1308171 |
| ENSRNOG00000006353 | 5.389773 | 1.018784 | 2.403376 | 6.46E-08 | 1.27E-06 | up | Large2 |
| ENSRNOG00000018454 | 17.69603 | 3.373459 | 2.391125 | 9.12E-13 | 3.37E-11 | up | Apoe |
| ENSRNOG00000047786 | 416.8117 | 79.91071 | 2.382935 | 1.82E-10 | 5.21E-09 | up | Ogn |
| ENSRNOG00000051158 | 13.41957 | 2.613924 | 2.360049 | 1.29E-13 | 5.21E-12 | up | Cfb |
| ENSRNOG00000014034 | 5.182753 | 1.018034 | 2.347933 | 1.55E-18 | 9.94E-17 | up | Olfml2a |
| ENSRNOG00000021086 | 7.054899 | 1.400223 | 2.332969 | 1.65E-11 | 5.35E-10 | up | Dtx4 |
| ENSRNOG00000060237 | 10.18908 | 2.034923 | 2.323978 | 1.88E-24 | 1.80E-22 | up | Inhbb |
| ENSRNOG00000012302 | 34.1702 | 6.844324 | 2.319759 | 7.09E-36 | 1.39E-33 | up | Gucy1a3 |
| ENSRNOG00000000413 | 194.0417 | 40.20762 | 2.270826 | 6.96E-60 | 4.68E-57 | up | Pln |
| ENSRNOG00000029141 | 13.62812 | 2.825165 | 2.27018 | 2.72E-16 | 1.41E-14 | up | Trabd2b |
| ENSRNOG00000004753 | 5.350697 | 1.125067 | 2.249716 | 0.000302 | 0.002883 | up | Napb |
| ENSRNOG00000004317 | 11.553 | 2.448694 | 2.238183 | 7.49E-20 | 5.31E-18 | up | Vipr2 |
| ENSRNOG00000042939 | 14.83506 | 3.247391 | 2.191658 | 6.26E-10 | 1.66E-08 | up | Nrip2 |
| ENSRNOG00000016496 | 12.69655 | 2.80688 | 2.177397 | 6.60E-16 | 3.34E-14 | up | Ctsc |
| ENSRNOG00000016957 | 2930.006 | 653.5042 | 2.164635 | 6.38E-97 | 1.97E-93 | up | Igfbp2 |
| ENSRNOG00000054420 | 18.40548 | 4.109636 | 2.163053 | 1.70E-25 | 1.68E-23 | up | Vdr |
| ENSRNOG00000053086 | 51.43395 | 11.69736 | 2.136538 | 6.88E-20 | 4.90E-18 | up | Selenop |
| ENSRNOG00000011238 | 25.74686 | 5.861468 | 2.135062 | 3.07E-22 | 2.49E-20 | up | Tiparp |
| ENSRNOG00000000728 | 10.90272 | 2.533488 | 2.105492 | 3.06E-08 | 6.33E-07 | up | Clic2 |
| ENSRNOG00000036960 | 7.004666 | 1.637379 | 2.096928 | 5.19E-20 | 3.77E-18 | up | Abcc9 |
| ENSRNOG00000014021 | 15.46473 | 3.615454 | 2.096733 | 5.84E-15 | 2.73E-13 | up | Matn4 |
| ENSRNOG00000016278 | 7.473757 | 1.757367 | 2.088418 | 0.007801 | 0.045365 | up | Ccl17 |
| ENSRNOG00000036301 | 1736.484 | 413.9646 | 2.068589 | 0.008035 | 0.046425 | up | AC128792.1 |
| ENSRNOG00000006649 | 21.1735 | 5.143656 | 2.041394 | 7.88E-27 | 8.78E-25 | up | Thrb |
| ENSRNOG00000040287 | 1749.862 | 430.9994 | 2.021483 | 2.63E-84 | 3.70E-81 | up | Cyp1b1 |
| ENSRNOG00000013925 | 15.66885 | 3.885026 | 2.011903 | 5.27E-17 | 2.87E-15 | up | Nox4 |
| ENSRNOG00000005592 | 5.226839 | 1.298041 | 2.009603 | 3.61E-13 | 1.40E-11 | up | Brinp2 |
| ENSRNOG00000009033 | 11.02416 | 2.756388 | 1.999818 | 3.96E-29 | 5.28E-27 | up | Cntn2 |
| ENSRNOG00000012772 | 1347.132 | 341.7409 | 1.978916 | 1.87E-78 | 2.06E-75 | up | Nqo1 |
| ENSRNOG00000012721 | 14.1824 | 3.645734 | 1.959821 | 1.40E-18 | 9.08E-17 | up | Ednra |
| ENSRNOG00000016245 | 6.949832 | 1.794411 | 1.953468 | 9.11E-09 | 2.09E-07 | up | Neto2 |
| ENSRNOG00000008758 | 109.4666 | 28.56394 | 1.938223 | 4.46E-36 | 8.84E-34 | up | Tspan18 |
| ENSRNOG00000052424 | 12.57107 | 3.363273 | 1.90217 | 2.21E-45 | 6.43E-43 | up | RGD1306556 |
| ENSRNOG00000030210 | 88.95653 | 23.82919 | 1.900371 | 6.17E-37 | 1.31E-34 | up | Fndc1 |
| ENSRNOG00000014508 | 38.79543 | 10.48065 | 1.888158 | 1.23E-12 | 4.46E-11 | up | Mgll |
| ENSRNOG00000005679 | 17.10032 | 4.649472 | 1.878884 | 5.09E-10 | 1.37E-08 | up | Fap |
| ENSRNOG00000026941 | 11.63986 | 3.169567 | 1.876716 | 3.77E-21 | 2.91E-19 | up | Tril |
| ENSRNOG00000024230 | 12.28018 | 3.349171 | 1.874457 | 7.38E-13 | 2.76E-11 | up | Tnfaip8l3 |
| ENSRNOG00000032150 | 5.349876 | 1.505676 | 1.829093 | 1.88E-11 | 6.03E-10 | up | Adcy2 |
| ENSRNOG00000017073 | 150.0137 | 42.2968 | 1.826474 | 9.10E-39 | 2.07E-36 | up | Car9 |
| ENSRNOG00000002947 | 28.44287 | 8.07553 | 1.816438 | 3.84E-16 | 1.98E-14 | up | Dpt |
| ENSRNOG00000029148 | 19.21183 | 5.508735 | 1.802202 | 2.07E-24 | 1.96E-22 | up | Pdgfd |
| ENSRNOG00000061530 | 5.181561 | 1.554729 | 1.736724 | 1.95E-05 | 0.000243 | up | Fgf16 |
| ENSRNOG00000060773 | 33.72125 | 10.31151 | 1.709403 | 1.36E-22 | 1.15E-20 | up | Sertad4 |
| ENSRNOG00000006931 | 9.479318 | 2.903675 | 1.706903 | 1.10E-11 | 3.64E-10 | up | Eepd1 |
| ENSRNOG00000001295 | 37.98725 | 11.95585 | 1.667798 | 5.20E-10 | 1.40E-08 | up | S100b |
| ENSRNOG00000024101 | 15.21736 | 4.811062 | 1.661291 | 2.38E-14 | 1.04E-12 | up | Phkb |
| ENSRNOG00000057078 | 79.74488 | 25.25742 | 1.658685 | 1.61E-25 | 1.62E-23 | up | Ddit4 |
| ENSRNOG00000011526 | 34.40114 | 10.8981 | 1.65838 | 6.84E-38 | 1.49E-35 | up | Pcsk6 |
| ENSRNOG00000016769 | 7.877942 | 2.511306 | 1.649381 | 2.90E-05 | 0.000349 | up | Rab38 |
| ENSRNOG00000018859 | 21.54721 | 6.888035 | 1.645337 | 3.10E-14 | 1.33E-12 | up | Pik3ip1 |
| ENSRNOG00000005621 | 11.64561 | 3.74147 | 1.638109 | 1.02E-07 | 1.92E-06 | up | Gxylt2 |
| ENSRNOG00000018054 | 6.278847 | 2.022746 | 1.634185 | 3.73E-05 | 0.000437 | up | F2rl2 |
| ENSRNOG00000059326 | 7.053917 | 2.274892 | 1.632627 | 4.11E-13 | 1.59E-11 | up | Abca9 |
| ENSRNOG00000018233 | 1721.405 | 556.1522 | 1.630035 | 7.70E-39 | 1.80E-36 | up | Gas6 |
| ENSRNOG00000013717 | 23.23172 | 7.53936 | 1.623582 | 6.25E-14 | 2.59E-12 | up | Bmp6 |
| ENSRNOG00000007319 | 70.22747 | 23.11392 | 1.603274 | 1.03E-27 | 1.27E-25 | up | Trib3 |
| ENSRNOG00000006623 | 86.26916 | 28.6464 | 1.590491 | 2.20E-17 | 1.25E-15 | up | Cd302 |
| ENSRNOG00000023148 | 367.0023 | 122.4171 | 1.583984 | 2.72E-29 | 3.73E-27 | up | Col11a1 |
| ENSRNOG00000028624 | 19.35833 | 6.544803 | 1.564533 | 1.69E-26 | 1.86E-24 | up | Kif26b |
| ENSRNOG00000020467 | 85.49437 | 29.07458 | 1.556071 | 1.25E-11 | 4.10E-10 | up | Nrep |
| ENSRNOG00000007081 | 41.50673 | 14.17017 | 1.550489 | 3.00E-27 | 3.54E-25 | up | Xdh |
| ENSRNOG00000047367 | 6.114247 | 2.088248 | 1.549882 | 1.37E-08 | 3.00E-07 | up | Card14 |
| ENSRNOG00000007529 | 6.460749 | 2.222784 | 1.539334 | 4.37E-06 | 6.19E-05 | up | Bmf |
| ENSRNOG00000010840 | 46.1877 | 15.92709 | 1.536026 | 4.90E-27 | 5.65E-25 | up | Adamtsl3 |
| ENSRNOG00000022941 | 39.99125 | 13.82268 | 1.532647 | 4.35E-05 | 0.000502 | up | LOC102549852 |
| ENSRNOG00000029598 | 15.31227 | 5.317607 | 1.525839 | 1.78E-09 | 4.47E-08 | up | Robo2 |
| ENSRNOG00000015594 | 9.640853 | 3.357768 | 1.521658 | 8.77E-08 | 1.68E-06 | up | Rftn2 |
| ENSRNOG00000013954 | 24.00604 | 8.361585 | 1.521549 | 1.91E-19 | 1.31E-17 | up | Alpl |
| ENSRNOG00000005574 | 9.073263 | 3.178009 | 1.513498 | 4.29E-11 | 1.33E-09 | up | Adamts8 |
| ENSRNOG00000010260 | 8.645316 | 3.031222 | 1.512019 | 5.85E-20 | 4.21E-18 | up | Dixdc1 |
| ENSRNOG00000002381 | 17.48552 | 6.134734 | 1.511088 | 1.84E-11 | 5.93E-10 | up | Bmp3 |
| ENSRNOG00000003134 | 5.239943 | 1.839353 | 1.510353 | 1.39E-07 | 2.57E-06 | up | Slc4a4 |
| ENSRNOG00000002053 | 5.42721 | 1.911002 | 1.505882 | 1.44E-13 | 5.75E-12 | up | Fras1 |
| ENSRNOG00000016695 | 1172.925 | 414.0097 | 1.502374 | 8.13E-44 | 2.28E-41 | up | Mmp2 |
| ENSRNOG00000019184 | 265.4818 | 93.76875 | 1.501434 | 2.04E-23 | 1.82E-21 | up | Npr3 |
| ENSRNOG00000015749 | 10.12359 | 3.5922 | 1.494781 | 0.000363 | 0.003393 | up | Nme3 |
| ENSRNOG00000007743 | 22.16426 | 7.870446 | 1.493718 | 3.70E-09 | 9.00E-08 | up | Mgst1 |
| ENSRNOG00000021916 | 19.65411 | 7.012652 | 1.486799 | 1.51E-19 | 1.05E-17 | up | Slc16a12 |
| ENSRNOG00000033697 | 41.95929 | 15.12819 | 1.471751 | 1.95E-17 | 1.13E-15 | up | Casp4 |
| ENSRNOG00000046660 | 47.29268 | 17.07374 | 1.469838 | 1.67E-18 | 1.07E-16 | up | Gpc6 |
| ENSRNOG00000058609 | 49.38551 | 17.88745 | 1.46514 | 1.64E-14 | 7.30E-13 | up | Palmd |
| ENSRNOG00000026605 | 120.7177 | 44.52814 | 1.438848 | 2.04E-18 | 1.28E-16 | up | Ifi27l2b |
| ENSRNOG00000020349 | 6.857804 | 2.535258 | 1.435614 | 0.000155 | 0.001591 | up | Rab3il1 |
| ENSRNOG00000062155 | 10.96986 | 4.055783 | 1.435493 | 0.001115 | 0.009032 | up | Rn60_1_2212.3 |
| ENSRNOG00000021840 | 8.99707 | 3.344414 | 1.427702 | 2.18E-12 | 7.67E-11 | up | Cped1 |
| ENSRNOG00000024294 | 53.40711 | 19.8942 | 1.424684 | 5.07E-10 | 1.37E-08 | up | AABR07019083.1 |
| ENSRNOG00000014532 | 90.57859 | 33.82366 | 1.421137 | 0.000597 | 0.00523 | up | Lbp |
| ENSRNOG00000055567 | 8.489424 | 3.18291 | 1.41532 | 5.85E-07 | 9.69E-06 | up | Fmnl2 |
| ENSRNOG00000004554 | 14.42497 | 5.412055 | 1.41432 | 4.04E-06 | 5.77E-05 | up | Dcn |
| ENSRNOG00000001627 | 1229.553 | 462.1217 | 1.411789 | 3.53E-36 | 7.16E-34 | up | Abi3bp |
| ENSRNOG00000013240 | 9.812213 | 3.700265 | 1.40695 | 1.14E-06 | 1.79E-05 | up | Ptger4 |
| ENSRNOG00000019270 | 33.43934 | 12.73597 | 1.392637 | 2.95E-22 | 2.41E-20 | up | P2ry6 |
| ENSRNOG00000016752 | 27.80146 | 10.68643 | 1.379381 | 7.78E-12 | 2.60E-10 | up | Crispld2 |
| ENSRNOG00000020332 | 6.897115 | 2.660799 | 1.374134 | 0.001308 | 0.010321 | up | Tnnt3 |
| ENSRNOG00000032410 | 8.280262 | 3.206777 | 1.368552 | 1.25E-07 | 2.32E-06 | up | Mtus2 |
| ENSRNOG00000028856 | 5.914914 | 2.297251 | 1.364449 | 8.93E-08 | 1.71E-06 | up | Pknox2 |
| ENSRNOG00000048389 | 22.7395 | 8.919857 | 1.350108 | 0.001748 | 0.013213 | up | Fgf18 |
| ENSRNOG00000030478 | 923.4941 | 365.5118 | 1.337185 | 7.79E-26 | 8.14E-24 | up | AY172581.9 |
| ENSRNOG00000007032 | 7.805649 | 3.100087 | 1.33221 | 3.29E-06 | 4.77E-05 | up | Amigo2 |
| ENSRNOG00000012660 | 11839.19 | 4708.904 | 1.330108 | 6.38E-05 | 0.000712 | up | Postn |
| ENSRNOG00000014964 | 21.1682 | 8.465382 | 1.322251 | 5.27E-07 | 8.76E-06 | up | Hp |
| ENSRNOG00000011973 | 6.046237 | 2.42518 | 1.317946 | 0.004049 | 0.026661 | up | Il7 |
| ENSRNOG00000015354 | 18.58776 | 7.483106 | 1.312644 | 1.00E-12 | 3.69E-11 | up | Aox1 |
| ENSRNOG00000004757 | 23.47916 | 9.45448 | 1.312311 | 1.64E-06 | 2.47E-05 | up | Tmem158 |
| ENSRNOG00000018962 | 36.46025 | 14.81072 | 1.299682 | 1.66E-12 | 5.90E-11 | up | Ctf1 |
| ENSRNOG00000010658 | 16.13154 | 6.582817 | 1.293107 | 2.52E-07 | 4.40E-06 | up | LOC103691744 |
| ENSRNOG00000037838 | 5.334412 | 2.195598 | 1.280716 | 0.000427 | 0.003904 | up | RGD1560455 |
| ENSRNOG00000011076 | 25.76448 | 10.63515 | 1.276543 | 1.61E-15 | 7.71E-14 | up | Ank2 |
| ENSRNOG00000009514 | 51.75012 | 21.47326 | 1.269021 | 1.43E-10 | 4.14E-09 | up | Mme |
| ENSRNOG00000019822 | 238.6273 | 99.42395 | 1.263094 | 4.03E-26 | 4.23E-24 | up | Gadd45b |
| ENSRNOG00000003687 | 12.45033 | 5.203783 | 1.258552 | 0.000549 | 0.00487 | up | Rgs2 |
| ENSRNOG00000000815 | 47.58602 | 19.97781 | 1.252139 | 3.00E-11 | 9.43E-10 | up | Smpdl3a |
| ENSRNOG00000016460 | 57.6416 | 24.23313 | 1.25013 | 1.90E-14 | 8.40E-13 | up | Clu |
| ENSRNOG00000016479 | 7.063457 | 2.999696 | 1.235558 | 1.32E-07 | 2.45E-06 | up | Plekhg4 |
| ENSRNOG00000015880 | 74.60794 | 31.91137 | 1.225259 | 4.42E-24 | 4.14E-22 | up | Dpep1 |
| ENSRNOG00000028619 | 12.77546 | 5.509127 | 1.21348 | 6.01E-08 | 1.19E-06 | up | Hoxc8 |
| ENSRNOG00000003183 | 16.74258 | 7.229576 | 1.211539 | 2.98E-13 | 1.17E-11 | up | Fmod |
| ENSRNOG00000030486 | 6.13874 | 2.65235 | 1.210671 | 0.000114 | 0.001198 | up | Prdm6 |
| ENSRNOG00000011161 | 7.769563 | 3.362268 | 1.208399 | 9.78E-09 | 2.22E-07 | up | Slc2a12 |
| ENSRNOG00000042960 | 244.4832 | 106.8494 | 1.194157 | 3.14E-11 | 9.81E-10 | up | Rgcc |
| ENSRNOG00000010841 | 400.2023 | 175.6379 | 1.188125 | 1.00E-24 | 9.67E-23 | up | Col8a2 |
| ENSRNOG00000048961 | 6.295015 | 2.764137 | 1.187381 | 0.001307 | 0.01032 | up | Bhlhe41 |
| ENSRNOG00000004516 | 459.0493 | 201.7621 | 1.185994 | 1.27E-17 | 7.49E-16 | up | Itgbl1 |
| ENSRNOG00000028390 | 23.85049 | 10.52276 | 1.180506 | 8.22E-13 | 3.06E-11 | up | Hhipl1 |
| ENSRNOG00000046330 | 19.13817 | 8.447187 | 1.17991 | 3.51E-06 | 5.07E-05 | up | Rnf130 |
| ENSRNOG00000004303 | 14.08093 | 6.23237 | 1.17589 | 9.87E-08 | 1.87E-06 | up | Timp3 |
| ENSRNOG00000011032 | 15.94333 | 7.070357 | 1.173098 | 9.29E-15 | 4.26E-13 | up | Lhfpl2 |
| ENSRNOG00000010635 | 23.38418 | 10.39565 | 1.169553 | 2.71E-09 | 6.73E-08 | up | Igfbp4 |
| ENSRNOG00000011895 | 13.23353 | 5.888546 | 1.168214 | 0.001902 | 0.014145 | up | LOC691143 |
| ENSRNOG00000057089 | 7.39144 | 3.294129 | 1.165958 | 0.000331 | 0.003126 | up | LOC103691744 |
| ENSRNOG00000010529 | 2398.809 | 1081.194 | 1.149693 | 5.61E-35 | 1.03E-32 | up | Thbs2 |
| ENSRNOG00000003479 | 16.26567 | 7.331716 | 1.149607 | 1.86E-11 | 5.96E-10 | up | Rnf150 |
| ENSRNOG00000019142 | 66.61263 | 30.02705 | 1.149533 | 1.05E-15 | 5.15E-14 | up | Fas |
| ENSRNOG00000008012 | 85.40464 | 38.71563 | 1.141398 | 6.48E-15 | 3.00E-13 | up | Abcb1a |
| ENSRNOG00000008757 | 16.05708 | 7.284519 | 1.140304 | 8.01E-07 | 1.29E-05 | up | Tmem218 |
| ENSRNOG00000042556 | 11.86853 | 5.404442 | 1.134923 | 0.001044 | 0.008522 | up | Bmyc |
| ENSRNOG00000053201 | 6.558606 | 2.99036 | 1.13307 | 6.13E-05 | 0.000688 | up | Gpcpd1 |
| ENSRNOG00000057556 | 125.6874 | 57.55177 | 1.126908 | 8.68E-28 | 1.09E-25 | up | Pdzrn3 |
| ENSRNOG00000017193 | 9.560327 | 4.393574 | 1.121665 | 3.80E-06 | 5.46E-05 | up | Lingo1 |
| ENSRNOG00000011971 | 119.9378 | 55.13934 | 1.121132 | 5.84E-14 | 2.43E-12 | up | C1s |
| ENSRNOG00000006631 | 5.76655 | 2.655733 | 1.118598 | 4.46E-06 | 6.28E-05 | up | Sema3e |
| ENSRNOG00000013663 | 8.847743 | 4.08729 | 1.114165 | 1.67E-05 | 0.00021 | up | Tmem86a |
| ENSRNOG00000011016 | 44.53681 | 20.58865 | 1.113149 | 1.06E-15 | 5.21E-14 | up | Slc7a2 |
| ENSRNOG00000016874 | 30.02601 | 14.00471 | 1.1003 | 7.23E-18 | 4.43E-16 | up | Zfp521 |
| ENSRNOG00000007964 | 12.90663 | 6.031888 | 1.097431 | 0.000181 | 0.00183 | up | Tp53inp1 |
| ENSRNOG00000002158 | 11.37058 | 5.316244 | 1.096826 | 2.63E-06 | 3.88E-05 | up | Ibsp |
| ENSRNOG00000010832 | 252.2101 | 118.0382 | 1.095372 | 8.97E-17 | 4.78E-15 | up | Pdgfrl |
| ENSRNOG00000007370 | 12.79367 | 6.009631 | 1.090082 | 1.46E-12 | 5.22E-11 | up | Rnf144a |
| ENSRNOG00000033970 | 7.480914 | 3.529159 | 1.08389 | 0.001147 | 0.009253 | up | Moap1 |
| ENSRNOG00000027245 | 25.4719 | 12.01988 | 1.083484 | 1.44E-12 | 5.17E-11 | up | Tdrp |
| ENSRNOG00000002372 | 15.78375 | 7.458746 | 1.081435 | 2.17E-06 | 3.25E-05 | up | Sgcd |
| ENSRNOG00000005348 | 5.09609 | 2.409394 | 1.08072 | 0.00013 | 0.001357 | up | Pamr1 |
| ENSRNOG00000047928 | 18.60534 | 8.799487 | 1.080225 | 0.004883 | 0.03112 | up | AABR07048653.1 |
| ENSRNOG00000000825 | 6.072528 | 2.890588 | 1.070934 | 0.00124 | 0.009887 | up | Fam26e |
| ENSRNOG00000021129 | 39.35826 | 18.77228 | 1.068062 | 1.21E-08 | 2.70E-07 | up | RGD1308428 |
| ENSRNOG00000061031 | 86.21189 | 41.73458 | 1.046644 | 5.94E-12 | 2.02E-10 | up | Fzd8 |
| ENSRNOG00000001953 | 6.122815 | 2.965722 | 1.045812 | 0.001138 | 0.009201 | up | Bace2 |
| ENSRNOG00000043866 | 560.4559 | 271.4757 | 1.045778 | 1.36E-19 | 9.50E-18 | up | AY172581.24 |
| ENSRNOG00000000657 | 104.2317 | 50.65066 | 1.041141 | 9.87E-12 | 3.26E-10 | up | Nek7 |
| ENSRNOG00000059244 | 87.20661 | 42.45052 | 1.038655 | 6.27E-12 | 2.12E-10 | up | LOC100909849 |
| ENSRNOG00000026128 | 7.852514 | 3.836184 | 1.033483 | 5.92E-07 | 9.80E-06 | up | Cpne8 |
| ENSRNOG00000004210 | 232.1796 | 113.4868 | 1.032717 | 5.74E-22 | 4.58E-20 | up | Osr1 |
| ENSRNOG00000018865 | 103.8141 | 51.07388 | 1.023344 | 2.17E-23 | 1.93E-21 | up | Adamts12 |
| ENSRNOG00000022812 | 10.02132 | 4.963127 | 1.013751 | 2.40E-05 | 0.000295 | up | Ercc5 |
| ENSRNOG00000002052 | 2127.101 | 1054.083 | 1.012901 | 1.06E-25 | 1.07E-23 | up | Ccdc80 |
| ENSRNOG00000020580 | 7.175924 | 3.556685 | 1.012631 | 0.000914 | 0.00756 | up | LOC500956 |
| ENSRNOG00000015904 | 1702.131 | 847.0849 | 1.006763 | 9.81E-26 | 1.01E-23 | up | Wfdc1 |
| ENSRNOG00000010695 | 35.75132 | 17.79336 | 1.006658 | 1.26E-10 | 3.67E-09 | up | Pdgfc |
| ENSRNOG00000021166 | 43.65677 | 87.34008 | -1.00044 | 5.27E-14 | 2.21E-12 | down | Ecm1 |
| ENSRNOG00000024043 | 4.825455 | 9.660339 | -1.00141 | 0.003051 | 0.02094 | down | Orc6 |
| ENSRNOG00000001214 | 29.45521 | 58.97641 | -1.00162 | 9.16E-09 | 2.10E-07 | down | Pfkl |
| ENSRNOG00000021750 | 57.67751 | 115.6335 | -1.00348 | 2.14E-08 | 4.59E-07 | down | Id1 |
| ENSRNOG00000002775 | 4.533517 | 9.091321 | -1.00386 | 2.84E-05 | 0.000343 | down | Npl |
| ENSRNOG00000050106 | 9.345553 | 18.7435 | -1.00404 | 6.96E-07 | 1.14E-05 | down | Rcc1 |
| ENSRNOG00000014187 | 14.54952 | 29.19046 | -1.00453 | 2.36E-09 | 5.87E-08 | down | Igf1r |
| ENSRNOG00000001859 | 38.12387 | 76.53286 | -1.00538 | 1.04E-09 | 2.70E-08 | down | Sdf2l1 |
| ENSRNOG00000001989 | 10.88752 | 21.87252 | -1.00644 | 1.36E-08 | 2.99E-07 | down | Alcam |
| ENSRNOG00000003546 | 310.3232 | 623.7546 | -1.00721 | 1.32E-10 | 3.84E-09 | down | Tnfrsf12a |
| ENSRNOG00000051854 | 12.83343 | 25.84465 | -1.00996 | 4.52E-11 | 1.40E-09 | down | Enpep |
| ENSRNOG00000010524 | 177.8471 | 358.2457 | -1.01031 | 7.44E-12 | 2.49E-10 | down | Cryab |
| ENSRNOG00000020676 | 116.8193 | 235.4897 | -1.01139 | 1.23E-12 | 4.46E-11 | down | Ppp1r14a |
| ENSRNOG00000055222 | 4.403057 | 8.876817 | -1.01154 | 0.005705 | 0.035408 | down | AC094212.1 |
| ENSRNOG00000010274 | 6.105109 | 12.32825 | -1.01388 | 8.18E-07 | 1.32E-05 | down | Smc4 |
| ENSRNOG00000025075 | 4.407013 | 8.903891 | -1.01463 | 0.001603 | 0.012284 | down | Relt |
| ENSRNOG00000061519 | 3.417838 | 6.906698 | -1.01491 | 0.000124 | 0.001293 | down | Asap2 |
| ENSRNOG00000019536 | 25.18372 | 50.9682 | -1.01711 | 2.67E-08 | 5.57E-07 | down | Smim3 |
| ENSRNOG00000010473 | 4.337379 | 8.783053 | -1.0179 | 4.71E-07 | 7.91E-06 | down | Cand2 |
| ENSRNOG00000020105 | 17.79929 | 36.05476 | -1.01837 | 5.00E-11 | 1.54E-09 | down | Klhl30 |
| ENSRNOG00000014571 | 66.69456 | 135.0986 | -1.01837 | 3.83E-12 | 1.32E-10 | down | Dbndd2 |
| ENSRNOG00000001645 | 28.22172 | 57.19366 | -1.01905 | 4.26E-16 | 2.19E-14 | down | Filip1l |
| ENSRNOG00000033528 | 69.65532 | 141.2141 | -1.01958 | 4.52E-10 | 1.23E-08 | down | Tll1 |
| ENSRNOG00000021447 | 9.558181 | 19.39014 | -1.02052 | 0.000228 | 0.002249 | down | Prr7 |
| ENSRNOG00000016099 | 37.21183 | 75.49372 | -1.0206 | 4.65E-10 | 1.26E-08 | down | Id4 |
| ENSRNOG00000031706 | 5.514685 | 11.18885 | -1.02071 | 0.005985 | 0.036836 | down | RGD1563601 |
| ENSRNOG00000015618 | 9.046702 | 18.36811 | -1.02174 | 2.92E-08 | 6.05E-07 | down | Wnt5a |
| ENSRNOG00000028677 | 8.145281 | 16.5597 | -1.02364 | 1.43E-07 | 2.62E-06 | down | LOC361346 |
| ENSRNOG00000005492 | 36.53846 | 74.47602 | -1.02736 | 2.93E-10 | 8.19E-09 | down | Hpcal1 |
| ENSRNOG00000048660 | 3.646907 | 7.440898 | -1.0288 | 0.000239 | 0.002347 | down | Gsg2 |
| ENSRNOG00000016818 | 6.937193 | 14.1558 | -1.02897 | 6.91E-08 | 1.35E-06 | down | Fgfr3 |
| ENSRNOG00000014786 | 8.825136 | 18.03017 | -1.03072 | 7.02E-07 | 1.15E-05 | down | Ccne1 |
| ENSRNOG00000045892 | 55.18294 | 112.7661 | -1.03104 | 2.88E-18 | 1.81E-16 | down | Cfl2 |
| ENSRNOG00000030568 | 5.084399 | 10.39244 | -1.03139 | 1.33E-08 | 2.92E-07 | down | Rgs12 |
| ENSRNOG00000004373 | 24.20607 | 49.53598 | -1.03311 | 4.66E-08 | 9.30E-07 | down | Ddx39a |
| ENSRNOG00000010058 | 23.51743 | 48.20137 | -1.03534 | 2.78E-08 | 5.79E-07 | down | Spry2 |
| ENSRNOG00000018052 | 24.89188 | 51.0671 | -1.03672 | 3.01E-08 | 6.23E-07 | down | Cnksr3 |
| ENSRNOG00000058329 | 114.0912 | 234.283 | -1.03806 | 2.70E-16 | 1.41E-14 | down | Prrx2 |
| ENSRNOG00000028733 | 5.673563 | 11.6556 | -1.0387 | 0.001461 | 0.011377 | down | Prkar1b |
| ENSRNOG00000019445 | 9.409493 | 19.33881 | -1.03931 | 2.05E-06 | 3.07E-05 | down | Msln |
| ENSRNOG00000020771 | 42.15641 | 86.81575 | -1.0422 | 8.55E-07 | 1.37E-05 | down | RGD1561590 |
| ENSRNOG00000007541 | 23.55287 | 48.53347 | -1.04308 | 6.21E-11 | 1.90E-09 | down | Fhl3 |
| ENSRNOG00000020882 | 14.66049 | 30.21757 | -1.04345 | 2.19E-08 | 4.70E-07 | down | Shkbp1 |
| ENSRNOG00000014524 | 22.22381 | 45.89898 | -1.04636 | 2.93E-10 | 8.19E-09 | down | S1pr3 |
| ENSRNOG00000005041 | 122.7146 | 253.5153 | -1.04677 | 7.03E-17 | 3.77E-15 | down | Crip2 |
| ENSRNOG00000017786 | 122.0693 | 252.9356 | -1.05107 | 6.74E-09 | 1.56E-07 | down | Acta1 |
| ENSRNOG00000005788 | 2.490981 | 5.175238 | -1.05491 | 4.33E-06 | 6.15E-05 | down | Cdk5rap2 |
| ENSRNOG00000013328 | 29.06301 | 60.47669 | -1.05719 | 1.81E-07 | 3.24E-06 | down | Rbpms |
| ENSRNOG00000004577 | 53.50595 | 111.346 | -1.05728 | 6.03E-17 | 3.26E-15 | down | Fez2 |
| ENSRNOG00000020441 | 6.951902 | 14.48417 | -1.059 | 6.30E-07 | 1.03E-05 | down | Wnk4 |
| ENSRNOG00000056836 | 341.2598 | 712.7779 | -1.06258 | 5.32E-17 | 2.89E-15 | down | Cav1 |
| ENSRNOG00000008243 | 2.908401 | 6.077911 | -1.06335 | 0.001813 | 0.013626 | down | Slc43a1 |
| ENSRNOG00000008932 | 3.753163 | 7.84941 | -1.06448 | 3.09E-06 | 4.50E-05 | down | Ncapd3 |
| ENSRNOG00000001816 | 8.689755 | 18.21677 | -1.06788 | 1.50E-05 | 0.00019 | down | Rfc4 |
| ENSRNOG00000004208 | 76.42751 | 160.3793 | -1.06932 | 6.52E-13 | 2.46E-11 | down | Crim1 |
| ENSRNOG00000000563 | 3.441035 | 7.224946 | -1.07014 | 2.14E-06 | 3.21E-05 | down | Adamts14 |
| ENSRNOG00000029441 | 15.92853 | 33.45445 | -1.07058 | 2.94E-11 | 9.26E-10 | down | Klhl2 |
| ENSRNOG00000053468 | 471.0836 | 990.0631 | -1.07154 | 3.58E-18 | 2.22E-16 | down | Tuba1b |
| ENSRNOG00000056756 | 288.6952 | 607.3533 | -1.07299 | 3.42E-15 | 1.62E-13 | down | Actn1 |
| ENSRNOG00000057509 | 14.68177 | 30.90641 | -1.07388 | 0.002964 | 0.020481 | down | AABR07019663.1 |
| ENSRNOG00000046791 | 6.738859 | 14.1981 | -1.07512 | 5.20E-06 | 7.20E-05 | down | Sh3rf3 |
| ENSRNOG00000018598 | 6.382374 | 13.44843 | -1.07527 | 7.41E-06 | 9.96E-05 | down | Ankrd1 |
| ENSRNOG00000050949 | 18.97934 | 40.03937 | -1.07699 | 1.48E-06 | 2.27E-05 | down | Ttc39c |
| ENSRNOG00000019681 | 2.456276 | 5.189255 | -1.07906 | 5.84E-05 | 0.000658 | down | Pold1 |
| ENSRNOG00000016454 | 7.180276 | 15.17207 | -1.07931 | 7.27E-08 | 1.42E-06 | down | Nasp |
| ENSRNOG00000009884 | 2310.616 | 4891.975 | -1.08214 | 1.40E-16 | 7.36E-15 | down | Lgals1 |
| ENSRNOG00000046560 | 52.75242 | 111.821 | -1.08388 | 6.56E-06 | 8.90E-05 | down | AC109096.1 |
| ENSRNOG00000018785 | 3.985369 | 8.45117 | -1.08444 | 0.000252 | 0.002464 | down | Slc16a13 |
| ENSRNOG00000016058 | 25.49598 | 54.35023 | -1.09202 | 1.42E-09 | 3.60E-08 | down | Kazald1 |
| ENSRNOG00000020904 | 5.767954 | 12.29987 | -1.09251 | 0.005297 | 0.03328 | down | Cdc42ep2 |
| ENSRNOG00000010306 | 93.13947 | 198.7505 | -1.09349 | 5.63E-12 | 1.93E-10 | down | H2afz |
| ENSRNOG00000018943 | 9.807498 | 20.93185 | -1.09374 | 0.000604 | 0.005279 | down | Tnnc1 |
| ENSRNOG00000007221 | 22.39785 | 47.80802 | -1.09389 | 9.76E-09 | 2.22E-07 | down | Dut |
| ENSRNOG00000013005 | 10.92416 | 23.32773 | -1.09452 | 1.17E-09 | 2.99E-08 | down | Rpa2 |
| ENSRNOG00000014361 | 18.18241 | 38.83209 | -1.09471 | 2.64E-07 | 4.58E-06 | down | Edn1 |
| ENSRNOG00000017369 | 19.79234 | 42.33579 | -1.09694 | 1.06E-08 | 2.38E-07 | down | Mustn1 |
| ENSRNOG00000019208 | 2.442461 | 5.226557 | -1.09753 | 0.003736 | 0.024846 | down | P2rx5 |
| ENSRNOG00000014320 | 43.47782 | 93.1458 | -1.09921 | 3.96E-19 | 2.65E-17 | down | Inhba |
| ENSRNOG00000031934 | 72.62468 | 155.6421 | -1.0997 | 1.84E-11 | 5.93E-10 | down | Enah |
| ENSRNOG00000045738 | 3.731645 | 7.998863 | -1.09998 | 0.004954 | 0.031484 | down | Ak4 |
| ENSRNOG00000001518 | 6.425248 | 13.7778 | -1.10052 | 6.25E-11 | 1.90E-09 | down | Itga6 |
| ENSRNOG00000054344 | 38.04261 | 81.83958 | -1.10518 | 4.86E-09 | 1.15E-07 | down | Ier2 |
| ENSRNOG00000013290 | 2.813875 | 6.0584 | -1.10638 | 4.15E-06 | 5.91E-05 | down | Nrip3 |
| ENSRNOG00000028543 | 15.32678 | 33.0907 | -1.11037 | 7.54E-10 | 1.99E-08 | down | AABR07052523.1 |
| ENSRNOG00000008376 | 9.914536 | 21.43242 | -1.11218 | 3.90E-10 | 1.07E-08 | down | Slc2a3 |
| ENSRNOG00000001828 | 26.71304 | 57.80997 | -1.11377 | 9.46E-14 | 3.89E-12 | down | Stk38l |
| ENSRNOG00000028650 | 18.75576 | 40.70739 | -1.11796 | 1.40E-13 | 5.61E-12 | down | Inf2 |
| ENSRNOG00000005798 | 14.9793 | 32.51452 | -1.11811 | 2.22E-08 | 4.75E-07 | down | Cav3 |
| ENSRNOG00000021248 | 10.1894 | 22.12272 | -1.11846 | 4.55E-11 | 1.41E-09 | down | Cdc25b |
| ENSRNOG00000004807 | 36.87228 | 80.0572 | -1.11849 | 5.09E-13 | 1.94E-11 | down | Arf2 |
| ENSRNOG00000016316 | 15.20438 | 33.16254 | -1.12507 | 6.57E-15 | 3.03E-13 | down | Mcm2 |
| ENSRNOG00000017037 | 10.83389 | 23.73255 | -1.13132 | 5.01E-07 | 8.37E-06 | down | Otud3 |
| ENSRNOG00000019582 | 6.712977 | 14.73267 | -1.13399 | 5.73E-10 | 1.52E-08 | down | Mthfd1l |
| ENSRNOG00000015625 | 5.349337 | 11.75044 | -1.13528 | 2.64E-06 | 3.90E-05 | down | MGC116202 |
| ENSRNOG00000024763 | 21.81165 | 48.02103 | -1.13857 | 1.13E-08 | 2.53E-07 | down | LOC685431 |
| ENSRNOG00000020906 | 5.660022 | 12.46274 | -1.13874 | 3.68E-05 | 0.000431 | down | Pola2 |
| ENSRNOG00000062181 | 2.913815 | 6.419954 | -1.13965 | 0.003609 | 0.024168 | down | Rn60_11_0620.2 |
| ENSRNOG00000003144 | 5.147671 | 11.3435 | -1.13987 | 3.30E-07 | 5.64E-06 | down | Gprc5c |
| ENSRNOG00000023226 | 436.8171 | 964.5111 | -1.14277 | 1.45E-22 | 1.23E-20 | down | S100a10 |
| ENSRNOG00000051915 | 6.230174 | 13.7758 | -1.14479 | 0.000179 | 0.001812 | down | Spred3 |
| ENSRNOG00000018371 | 187.2798 | 414.2327 | -1.14525 | 2.16E-14 | 9.43E-13 | down | Tubb6 |
| ENSRNOG00000049269 | 14.86653 | 32.88309 | -1.14528 | 2.04E-09 | 5.10E-08 | down | RGD1563294 |
| ENSRNOG00000021062 | 96.13146 | 212.6902 | -1.14567 | 3.89E-13 | 1.51E-11 | down | Fxyd5 |
| ENSRNOG00000012991 | 67.09311 | 148.5523 | -1.14673 | 6.26E-17 | 3.37E-15 | down | Adgra2 |
| ENSRNOG00000011815 | 6.87613 | 15.23927 | -1.14813 | 1.31E-08 | 2.88E-07 | down | Sgk1 |
| ENSRNOG00000017505 | 3.983848 | 8.830902 | -1.1484 | 4.35E-06 | 6.16E-05 | down | Npepo |
| ENSRNOG00000012868 | 8.672091 | 19.27954 | -1.15262 | 1.27E-13 | 5.14E-12 | down | Uaca |
| ENSRNOG00000054614 | 12.87736 | 28.6765 | -1.15503 | 2.81E-05 | 0.000339 | down | AABR07002779.1 |
| ENSRNOG00000016728 | 4.327619 | 9.659341 | -1.15835 | 5.79E-09 | 1.36E-07 | down | Tiam2 |
| ENSRNOG00000009683 | 142.1556 | 317.9677 | -1.16141 | 9.64E-26 | 1.00E-23 | down | Sdcbp |
| ENSRNOG00000015366 | 4.865759 | 10.89462 | -1.16288 | 0.0026 | 0.018314 | down | Neurl3 |
| ENSRNOG00000050792 | 11.3209 | 25.3993 | -1.1658 | 8.20E-08 | 1.58E-06 | down | Tnfaip6 |
| ENSRNOG00000007984 | 3.769527 | 8.480828 | -1.16982 | 5.12E-08 | 1.02E-06 | down | Amer1 |
| ENSRNOG00000006876 | 14.21803 | 32.00429 | -1.17054 | 3.25E-10 | 9.02E-09 | down | Msx1 |
| ENSRNOG00000003120 | 105.2084 | 237.0752 | -1.1721 | 1.96E-19 | 1.34E-17 | down | Prelp |
| ENSRNOG00000059870 | 2.693952 | 6.073402 | -1.17278 | 0.000212 | 0.00211 | down | Hoxa11 |
| ENSRNOG00000062143 | 7.315273 | 16.50223 | -1.17368 | 1.28E-05 | 0.000165 | down | LOC100911830 |
| ENSRNOG00000049298 | 11.48811 | 25.94819 | -1.17549 | 7.89E-07 | 1.28E-05 | down | LOC100909595 |
| ENSRNOG00000004680 | 3.836202 | 8.668069 | -1.17603 | 6.74E-11 | 2.02E-09 | down | Kif5c |
| ENSRNOG00000011647 | 2578.638 | 5826.956 | -1.17613 | 9.62E-28 | 1.20E-25 | down | S100a6 |
| ENSRNOG00000014838 | 12.43381 | 28.13365 | -1.17803 | 3.08E-06 | 4.49E-05 | down | Glipr2 |
| ENSRNOG00000003650 | 9.195067 | 20.88592 | -1.1836 | 3.32E-05 | 0.000394 | down | Nt5c |
| ENSRNOG00000025742 | 7.87856 | 17.95245 | -1.18818 | 6.19E-07 | 1.02E-05 | down | Lmnb2 |
| ENSRNOG00000001245 | 5.177369 | 11.79916 | -1.18839 | 9.17E-07 | 1.46E-05 | down | Pcbp3 |
| ENSRNOG00000011452 | 6.402546 | 14.61951 | -1.19118 | 3.48E-05 | 0.00041 | down | Aldoc |
| ENSRNOG00000016023 | 33.48411 | 76.51752 | -1.19231 | 6.29E-22 | 4.98E-20 | down | Kank1 |
| ENSRNOG00000007286 | 3.008477 | 6.876451 | -1.19263 | 5.00E-06 | 6.96E-05 | down | Mdm1 |
| ENSRNOG00000007393 | 18.74397 | 42.89433 | -1.19436 | 9.29E-18 | 5.57E-16 | down | Ndrg1 |
| ENSRNOG00000053884 | 4.154146 | 9.507305 | -1.19448 | 0.001668 | 0.012726 | down | Hoxa11 |
| ENSRNOG00000008051 | 16.24893 | 37.24807 | -1.19682 | 1.51E-12 | 5.39E-11 | down | Itpk1 |
| ENSRNOG00000020355 | 10.52139 | 24.14504 | -1.1984 | 2.22E-06 | 3.31E-05 | down | Twist2 |
| ENSRNOG00000010386 | 38.4265 | 88.36375 | -1.20135 | 1.06E-17 | 6.28E-16 | down | H2afx |
| ENSRNOG00000010716 | 4.951348 | 11.38764 | -1.20158 | 4.23E-06 | 6.02E-05 | down | Atoh8 |
| ENSRNOG00000019180 | 8.585531 | 19.77999 | -1.20406 | 8.78E-16 | 4.36E-14 | down | Acsl4 |
| ENSRNOG00000008885 | 16.81465 | 38.7429 | -1.20421 | 1.40E-06 | 2.16E-05 | down | Chst11 |
| ENSRNOG00000047864 | 4.421333 | 10.21206 | -1.20772 | 0.000583 | 0.005118 | down | RT1-DMa |
| ENSRNOG00000024008 | 4.541989 | 10.49662 | -1.20853 | 5.14E-08 | 1.02E-06 | down | Cdc25c |
| ENSRNOG00000050946 | 3.507315 | 8.11557 | -1.21033 | 0.000511 | 0.004571 | down | Fam110a |
| ENSRNOG00000010875 | 24.93258 | 57.73153 | -1.21133 | 1.17E-07 | 2.20E-06 | down | LOC691931 |
| ENSRNOG00000030118 | 262.8959 | 609.8184 | -1.21389 | 9.16E-18 | 5.51E-16 | down | Msn |
| ENSRNOG00000002215 | 14.37524 | 33.51708 | -1.22131 | 3.34E-17 | 1.87E-15 | down | Mylk |
| ENSRNOG00000009263 | 206.2087 | 481.4069 | -1.22315 | 2.06E-17 | 1.19E-15 | down | Ifi27 |
| ENSRNOG00000018567 | 57.66919 | 134.6665 | -1.22352 | 8.98E-23 | 7.76E-21 | down | Slc20a1 |
| ENSRNOG00000048704 | 6.398616 | 14.96047 | -1.22532 | 2.88E-05 | 0.000347 | down | RGD1562758 |
| ENSRNOG00000021098 | 4.944932 | 11.56995 | -1.22636 | 3.37E-07 | 5.76E-06 | down | Rasgrp2 |
| ENSRNOG00000007607 | 39.63675 | 92.86499 | -1.2283 | 8.97E-18 | 5.42E-16 | down | Nr4a1 |
| ENSRNOG00000016119 | 26.6007 | 62.39191 | -1.22989 | 2.75E-13 | 1.08E-11 | down | Fzd7 |
| ENSRNOG00000017164 | 33.27285 | 78.20224 | -1.23286 | 1.91E-26 | 2.08E-24 | down | Afap1l2 |
| ENSRNOG00000018384 | 56.51028 | 132.8558 | -1.23328 | 1.00E-15 | 4.97E-14 | down | Adam12 |
| ENSRNOG00000023643 | 2.648894 | 6.240369 | -1.23624 | 0.00041 | 0.003778 | down | Mmp17 |
| ENSRNOG00000031342 | 2.242762 | 5.287972 | -1.23744 | 0.00225 | 0.016305 | down | AABR07063082.1 |
| ENSRNOG00000049629 | 3.58729 | 8.486789 | -1.24232 | 0.000275 | 0.002657 | down | Reep2 |
| ENSRNOG00000056585 | 32.97154 | 78.10492 | -1.24419 | 1.50E-15 | 7.24E-14 | down | Fscn1 |
| ENSRNOG00000005450 | 5.848629 | 13.90017 | -1.24893 | 2.00E-05 | 0.000249 | down | Lsm11 |
| ENSRNOG00000009079 | 5.101185 | 12.13512 | -1.25028 | 1.06E-09 | 2.72E-08 | down | Prkar2b |
| ENSRNOG00000046368 | 3.70476 | 8.82113 | -1.25158 | 0.007794 | 0.045348 | down | Dusp14l1 |
| ENSRNOG00000021412 | 3.474709 | 8.286236 | -1.25382 | 1.11E-05 | 0.000145 | down | Slfn13 |
| ENSRNOG00000005809 | 3.823706 | 9.11876 | -1.25387 | 0.000282 | 0.002713 | down | Arhgdib |
| ENSRNOG00000030871 | 139.2536 | 332.9058 | -1.2574 | 1.64E-22 | 1.37E-20 | down | Calm2 |
| ENSRNOG00000002652 | 4.536941 | 10.8593 | -1.25914 | 4.26E-08 | 8.56E-07 | down | Rap1gap2 |
| ENSRNOG00000023546 | 396.5937 | 949.3022 | -1.25921 | 2.48E-20 | 1.82E-18 | down | Hspb1 |
| ENSRNOG00000018858 | 4.616356 | 11.06589 | -1.26129 | 0.002843 | 0.019775 | down | Myct1 |
| ENSRNOG00000030629 | 4.871908 | 11.71379 | -1.26565 | 0.00011 | 0.001164 | down | Camkmt |
| ENSRNOG00000008178 | 38.23537 | 91.96072 | -1.26611 | 1.47E-18 | 9.48E-17 | down | Nxn |
| ENSRNOG00000003669 | 8.991048 | 21.64669 | -1.26759 | 1.69E-12 | 5.98E-11 | down | Myocd |
| ENSRNOG00000001414 | 1238.045 | 2986.826 | -1.27055 | 8.68E-18 | 5.31E-16 | down | Serpine1 |
| ENSRNOG00000030091 | 11.64698 | 28.10506 | -1.27087 | 2.44E-10 | 6.91E-09 | down | Dusp14 |
| ENSRNOG00000005153 | 2.445324 | 5.902464 | -1.27129 | 1.11E-09 | 2.85E-08 | down | LOC690422 |
| ENSRNOG00000047206 | 3.874136 | 9.352423 | -1.27147 | 3.26E-07 | 5.60E-06 | down | LOC100911727 |
| ENSRNOG00000039415 | 17.76115 | 42.8871 | -1.27182 | 2.23E-17 | 1.27E-15 | down | Fchsd1 |
| ENSRNOG00000019440 | 5.460673 | 13.22954 | -1.27661 | 7.44E-06 | 9.98E-05 | down | Kcnn4 |
| ENSRNOG00000037225 | 8.510311 | 20.63828 | -1.27804 | 1.35E-07 | 2.50E-06 | down | Tyms |
| ENSRNOG00000001602 | 14.07217 | 34.1429 | -1.27874 | 1.44E-08 | 3.15E-07 | down | Ltn1 |
| ENSRNOG00000020733 | 3.308757 | 8.062257 | -1.28489 | 0.003952 | 0.026159 | down | Camp |
| ENSRNOG00000002926 | 53.40992 | 130.1772 | -1.2853 | 3.94E-17 | 2.18E-15 | down | Uap1 |
| ENSRNOG00000014193 | 5.60111 | 13.66828 | -1.28705 | 1.25E-10 | 3.65E-09 | down | Lig1 |
| ENSRNOG00000001830 | 4.453092 | 10.87501 | -1.28814 | 1.50E-05 | 0.00019 | down | Arntl2 |
| ENSRNOG00000009946 | 34.91957 | 85.28959 | -1.28833 | 9.93E-15 | 4.53E-13 | down | Ldlr |
| ENSRNOG00000001584 | 10.26892 | 25.13382 | -1.29135 | 7.20E-07 | 1.17E-05 | down | Map3k7cl |
| ENSRNOG00000055451 | 6.674456 | 16.3533 | -1.29286 | 8.95E-07 | 1.43E-05 | down | Gcnt4 |
| ENSRNOG00000006469 | 5.520656 | 13.53098 | -1.29335 | 5.14E-09 | 1.21E-07 | down | Cdk2 |
| ENSRNOG00000047102 | 3.38025 | 8.288374 | -1.29396 | 0.001006 | 0.008249 | down | Popdc3 |
| ENSRNOG00000011800 | 225.4342 | 553.0278 | -1.29465 | 1.09E-22 | 9.35E-21 | down | F3 |
| ENSRNOG00000009867 | 402.4244 | 990.2291 | -1.29904 | 5.51E-28 | 7.10E-26 | down | Tgfb3 |
| ENSRNOG00000037295 | 2.752493 | 6.799788 | -1.30475 | 0.002158 | 0.015686 | down | Poc1a |
| ENSRNOG00000038375 | 7.827109 | 19.42399 | -1.31129 | 6.44E-06 | 8.76E-05 | down | AABR07026311.1 |
| ENSRNOG00000050183 | 2.134294 | 5.303848 | -1.31328 | 0.004287 | 0.027886 | down | RT1-CE1 |
| ENSRNOG00000022523 | 2.145439 | 5.365317 | -1.32239 | 1.32E-05 | 0.00017 | down | Fkbp5 |
| ENSRNOG00000008937 | 327.8655 | 821.4274 | -1.32503 | 5.72E-27 | 6.51E-25 | down | Csrp1 |
| ENSRNOG00000009957 | 4.785804 | 12.05856 | -1.33322 | 3.27E-06 | 4.74E-05 | down | Slc25a13 |
| ENSRNOG00000007726 | 35.43724 | 89.46721 | -1.33609 | 2.92E-13 | 1.15E-11 | down | Mcam |
| ENSRNOG00000012183 | 14.14558 | 35.78972 | -1.33919 | 8.47E-13 | 3.15E-11 | down | Glrx |
| ENSRNOG00000015329 | 46.01951 | 116.4697 | -1.33964 | 1.25E-27 | 1.52E-25 | down | Kpna2 |
| ENSRNOG00000022101 | 2.851742 | 7.230025 | -1.34216 | 0.003512 | 0.023568 | down | Crabp2 |
| ENSRNOG00000046202 | 35.54828 | 90.18998 | -1.34319 | 1.15E-19 | 8.04E-18 | down | Metrnl |
| ENSRNOG00000060381 | 20.78064 | 52.81712 | -1.34577 | 3.61E-26 | 3.88E-24 | down | Col15a1 |
| ENSRNOG00000051952 | 17.9975 | 45.8072 | -1.34778 | 4.15E-15 | 1.96E-13 | down | Tes |
| ENSRNOG00000053592 | 4.818907 | 12.29624 | -1.35144 | 0.008308 | 0.047792 | down | AABR07050646.1 |
| ENSRNOG00000019202 | 24.16314 | 61.66721 | -1.3517 | 1.40E-18 | 9.08E-17 | down | PVR |
| ENSRNOG00000029212 | 26.49738 | 67.68295 | -1.35294 | 2.98E-31 | 4.43E-29 | down | Vcan |
| ENSRNOG00000004019 | 40.27857 | 102.9992 | -1.35455 | 2.40E-14 | 1.04E-12 | down | Phlda1 |
| ENSRNOG00000047854 | 3.475846 | 8.916826 | -1.35917 | 0.000219 | 0.002169 | down | LOC108348072 |
| ENSRNOG00000011817 | 45.82328 | 117.5752 | -1.35943 | 1.02E-15 | 5.02E-14 | down | Rpl22l1 |
| ENSRNOG00000028992 | 203.8321 | 523.067 | -1.35961 | 1.61E-20 | 1.20E-18 | down | Acan |
| ENSRNOG00000039197 | 19.6607 | 50.61701 | -1.36431 | 1.97E-23 | 1.77E-21 | down | LOC108348074 |
| ENSRNOG00000007905 | 49.97426 | 128.7402 | -1.36521 | 0.000468 | 0.004223 | down | Itga7 |
| ENSRNOG00000000566 | 3.833793 | 9.884271 | -1.36636 | 0.000235 | 0.002309 | down | Pcbd1 |
| ENSRNOG00000020480 | 5.906485 | 15.25811 | -1.3692 | 1.33E-13 | 5.37E-12 | down | Fads1 |
| ENSRNOG00000013391 | 11.70773 | 30.26121 | -1.37001 | 1.99E-27 | 2.40E-25 | down | Sorbs2 |
| ENSRNOG00000037113 | 9.691292 | 25.08921 | -1.37231 | 3.04E-09 | 7.47E-08 | down | Slfn2 |
| ENSRNOG00000011821 | 1490.376 | 3887.675 | -1.38323 | 5.41E-32 | 8.54E-30 | down | S100a4 |
| ENSRNOG00000037661 | 17.34971 | 45.28037 | -1.38397 | 1.65E-06 | 2.49E-05 | down | Tmsbl1 |
| ENSRNOG00000009204 | 2.157354 | 5.635758 | -1.38535 | 4.85E-07 | 8.14E-06 | down | Il17re |
| ENSRNOG00000031612 | 14.86301 | 38.89526 | -1.38787 | 1.42E-14 | 6.35E-13 | down | Gls2 |
| ENSRNOG00000013589 | 67.57124 | 177.2785 | -1.39154 | 2.96E-16 | 1.54E-14 | down | Cxcl12 |
| ENSRNOG00000001134 | 7.154788 | 18.82782 | -1.39589 | 2.57E-09 | 6.39E-08 | down | Rfc5 |
| ENSRNOG00000007281 | 38.23171 | 100.9306 | -1.40052 | 0.000201 | 0.00201 | down | Flnc |
| ENSRNOG00000018214 | 14.67627 | 38.83011 | -1.40369 | 2.49E-14 | 1.07E-12 | down | Bok |
| ENSRNOG00000007827 | 5.258023 | 13.95291 | -1.40797 | 5.29E-06 | 7.30E-05 | down | Cox4i2 |
| ENSRNOG00000027234 | 2.867531 | 7.637138 | -1.41322 | 9.33E-08 | 1.78E-06 | down | Zfp367 |
| ENSRNOG00000016110 | 6.053172 | 16.15013 | -1.41578 | 7.53E-09 | 1.74E-07 | down | Kcnk12 |
| ENSRNOG00000055305 | 8.698599 | 23.37488 | -1.4261 | 5.16E-15 | 2.43E-13 | down | Parvb |
| ENSRNOG00000045752 | 22.05361 | 59.43531 | -1.43031 | 1.22E-27 | 1.50E-25 | down | Rrm1 |
| ENSRNOG00000020552 | 10.79484 | 29.14068 | -1.43269 | 3.71E-08 | 7.56E-07 | down | Fosl1 |
| ENSRNOG00000001349 | 9.700132 | 26.20069 | -1.43353 | 4.95E-17 | 2.71E-15 | down | Mcm7 |
| ENSRNOG00000013179 | 22.04422 | 59.89728 | -1.44209 | 4.75E-13 | 1.83E-11 | down | Tinagl1 |
| ENSRNOG00000030058 | 5.694119 | 15.53977 | -1.44842 | 6.30E-11 | 1.91E-09 | down | LOC100359600 |
| ENSRNOG00000016538 | 29.97854 | 81.88596 | -1.44969 | 7.94E-27 | 8.78E-25 | down | Itga8 |
| ENSRNOG00000008956 | 9.908793 | 27.11613 | -1.45237 | 5.21E-13 | 1.98E-11 | down | Cdkn2c |
| ENSRNOG00000027592 | 1.884344 | 5.166126 | -1.45502 | 5.16E-05 | 0.000589 | down | Rerg |
| ENSRNOG00000024757 | 8.674961 | 23.8721 | -1.4604 | 2.71E-08 | 5.64E-07 | down | Endod1 |
| ENSRNOG00000013215 | 2.898862 | 7.983396 | -1.46152 | 2.97E-07 | 5.12E-06 | down | Dctd |
| ENSRNOG00000058186 | 5.562464 | 15.3358 | -1.46311 | 1.01E-14 | 4.58E-13 | down | Errfi1 |
| ENSRNOG00000037339 | 2.224079 | 6.135681 | -1.46402 | 1.48E-05 | 0.000188 | down | Siglec10 |
| ENSRNOG00000054524 | 2.424063 | 6.720884 | -1.47122 | 9.37E-07 | 1.48E-05 | down | Tnip3 |
| ENSRNOG00000001833 | 9.303364 | 26.2936 | -1.49889 | 3.48E-22 | 2.80E-20 | down | Mcm4 |
| ENSRNOG00000043199 | 18.73506 | 52.96969 | -1.49943 | 2.01E-11 | 6.42E-10 | down | Bves |
| ENSRNOG00000056298 | 4.303339 | 12.25952 | -1.51037 | 0.00043 | 0.003921 | down | AABR07044959.1 |
| ENSRNOG00000008936 | 3.032807 | 8.641237 | -1.51058 | 2.51E-11 | 7.93E-10 | down | Map3k6 |
| ENSRNOG00000048726 | 4.517878 | 12.93833 | -1.51793 | 0.00026 | 0.002529 | down | Clec2d |
| ENSRNOG00000030224 | 2.253891 | 6.456467 | -1.51833 | 0.000677 | 0.005832 | down | AABR07030729.1 |
| ENSRNOG00000046479 | 4.295854 | 12.3237 | -1.52042 | 2.32E-11 | 7.37E-10 | down | Chaf1a |
| ENSRNOG00000017386 | 2.139278 | 6.138523 | -1.52077 | 0.000135 | 0.0014 | down | Il11 |
| ENSRNOG00000008479 | 6.272218 | 18.13664 | -1.53186 | 9.72E-15 | 4.45E-13 | down | Slc8a1 |
| ENSRNOG00000007415 | 21.57699 | 62.56184 | -1.53579 | 2.69E-32 | 4.47E-30 | down | Ptgs1 |
| ENSRNOG00000048703 | 80.64666 | 234.4198 | -1.53941 | 1.12E-23 | 1.02E-21 | down | Fam150a |
| ENSRNOG00000024264 | 4.124486 | 11.99229 | -1.53982 | 3.40E-08 | 6.96E-07 | down | Amz1 |
| ENSRNOG00000012460 | 3.462637 | 10.1473 | -1.55115 | 6.26E-06 | 8.53E-05 | down | Cntf |
| ENSRNOG00000011167 | 1.721547 | 5.048095 | -1.55203 | 3.92E-09 | 9.48E-08 | down | Wdhd1 |
| ENSRNOG00000014530 | 2.407382 | 7.060972 | -1.5524 | 1.59E-19 | 1.09E-17 | down | Nav2 |
| ENSRNOG00000049743 | 49.09015 | 144.2068 | -1.55463 | 3.52E-25 | 3.44E-23 | down | NEWGENE_620381 |
| ENSRNOG00000003069 | 17.25457 | 50.72486 | -1.55571 | 1.71E-25 | 1.69E-23 | down | Cd38 |
| ENSRNOG00000040350 | 34.16542 | 101.3062 | -1.56811 | 0.000188 | 0.001897 | down | Mir675 |
| ENSRNOG00000022325 | 2.882176 | 8.550581 | -1.56886 | 6.67E-09 | 1.55E-07 | down | Smc2 |
| ENSRNOG00000026644 | 8.822641 | 26.21152 | -1.57092 | 2.51E-11 | 7.93E-10 | down | Glipr1 |
| ENSRNOG00000008641 | 8.765464 | 26.1844 | -1.57881 | 2.18E-10 | 6.20E-09 | down | Gnpnat1 |
| ENSRNOG00000011411 | 4.875148 | 14.57383 | -1.57986 | 3.61E-24 | 3.40E-22 | down | Adgrg6 |
| ENSRNOG00000025042 | 8.433795 | 25.22445 | -1.58057 | 8.56E-21 | 6.52E-19 | down | Pde3a |
| ENSRNOG00000000795 | 2.745939 | 8.21663 | -1.58125 | 2.50E-05 | 0.000305 | down | RT1-N3 |
| ENSRNOG00000036677 | 33.85984 | 101.4362 | -1.58293 | 0.000262 | 0.002546 | down | Slc16a3 |
| ENSRNOG00000018524 | 2.991616 | 8.963093 | -1.58307 | 1.58E-10 | 4.54E-09 | down | Ezr |
| ENSRNOG00000018944 | 3.80807 | 11.4424 | -1.58726 | 2.16E-12 | 7.60E-11 | down | Pank1 |
| ENSRNOG00000020531 | 4.775063 | 14.37667 | -1.59014 | 1.55E-12 | 5.55E-11 | down | Fen1 |
| ENSRNOG00000027736 | 174.5857 | 525.9885 | -1.5911 | 3.93E-26 | 4.16E-24 | down | Cnn1 |
| ENSRNOG00000057451 | 54.61746 | 165.3995 | -1.59852 | 9.79E-21 | 7.42E-19 | down | Itga5 |
| ENSRNOG00000058645 | 273.7033 | 832.3899 | -1.60465 | 2.17E-35 | 4.15E-33 | down | Tnc |
| ENSRNOG00000033433 | 8.190027 | 24.91572 | -1.60512 | 0.004776 | 0.030538 | down | Csrnp1 |
| ENSRNOG00000004841 | 2.983538 | 9.090826 | -1.60739 | 3.18E-13 | 1.24E-11 | down | Akap6 |
| ENSRNOG00000000017 | 4.822441 | 14.82996 | -1.62068 | 9.86E-09 | 2.23E-07 | down | Steap1 |
| ENSRNOG00000029399 | 4.997621 | 15.37679 | -1.62144 | 9.71E-10 | 2.52E-08 | down | Bcam |
| ENSRNOG00000022686 | 7.254227 | 22.40693 | -1.62705 | 7.07E-12 | 2.38E-10 | down | Zdhhc2 |
| ENSRNOG00000019810 | 1.794556 | 5.544083 | -1.62732 | 1.41E-05 | 0.00018 | down | Des |
| ENSRNOG00000020407 | 2.981292 | 9.220068 | -1.62884 | 3.68E-13 | 1.43E-11 | down | Atcay |
| ENSRNOG00000009951 | 6.829495 | 21.16323 | -1.63171 | 5.68E-14 | 2.37E-12 | down | Aif1l |
| ENSRNOG00000011654 | 2.640413 | 8.28861 | -1.65037 | 5.65E-12 | 1.93E-10 | down | Plk4 |
| ENSRNOG00000046333 | 4.808455 | 15.26182 | -1.66628 | 9.93E-07 | 1.57E-05 | down | Ablim1 |
| ENSRNOG00000027109 | 4.776099 | 15.1641 | -1.66676 | 9.49E-09 | 2.16E-07 | down | Odf3l1 |
| ENSRNOG00000006048 | 3.78392 | 12.04208 | -1.67013 | 1.21E-14 | 5.43E-13 | down | Ezh2 |
| ENSRNOG00000024243 | 60.87045 | 193.8784 | -1.67134 | 1.00E-38 | 2.24E-36 | down | Cadm4 |
| ENSRNOG00000016708 | 5.119357 | 16.46706 | -1.68555 | 2.15E-17 | 1.23E-15 | down | E2f1 |
| ENSRNOG00000003517 | 5.541279 | 17.87233 | -1.68944 | 6.39E-09 | 1.49E-07 | down | Tbx2 |
| ENSRNOG00000014137 | 2.06729 | 6.69909 | -1.69622 | 9.75E-08 | 1.85E-06 | down | Fbln1 |
| ENSRNOG00000033321 | 2.601226 | 8.437685 | -1.69766 | 0.000618 | 0.005381 | down | Hmgb2 |
| ENSRNOG00000019620 | 9.255978 | 30.17231 | -1.70477 | 2.08E-14 | 9.13E-13 | down | Pmf1 |
| ENSRNOG00000013306 | 5.024878 | 16.4062 | -1.70708 | 8.52E-15 | 3.92E-13 | down | Pcdh20 |
| ENSRNOG00000003464 | 1.934976 | 6.341041 | -1.7124 | 2.92E-10 | 8.19E-09 | down | Hid1 |
| ENSRNOG00000015365 | 6.799964 | 22.33922 | -1.71598 | 2.86E-29 | 3.87E-27 | down | Col4a3 |
| ENSRNOG00000055300 | 3.943844 | 13.01681 | -1.7227 | 5.41E-21 | 4.16E-19 | down | Ncapd2 |
| ENSRNOG00000049075 | 63.76349 | 210.4861 | -1.72292 | 7.38E-23 | 6.48E-21 | down | Fabp5 |
| ENSRNOG00000046521 | 4.918677 | 16.33018 | -1.7312 | 9.50E-11 | 2.80E-09 | down | LOC100911572 |
| ENSRNOG00000016846 | 1.978054 | 6.60459 | -1.73939 | 1.09E-12 | 3.99E-11 | down | Pik3cd |
| ENSRNOG00000002045 | 10.65292 | 35.57775 | -1.73973 | 6.33E-30 | 8.81E-28 | down | Anxa3 |
| ENSRNOG00000005650 | 4.540321 | 15.2322 | -1.74626 | 9.15E-12 | 3.04E-10 | down | Pgf |
| ENSRNOG00000001979 | 52.60799 | 177.0662 | -1.75093 | 1.98E-22 | 1.64E-20 | down | Rcan1 |
| ENSRNOG00000002436 | 12.93174 | 43.79376 | -1.75981 | 3.24E-31 | 4.77E-29 | down | Mmd |
| ENSRNOG00000018040 | 4.117584 | 13.99337 | -1.76487 | 1.33E-08 | 2.92E-07 | down | Gins4 |
| ENSRNOG00000012008 | 1.973763 | 6.73155 | -1.76999 | 0.007559 | 0.044248 | down | S100a3 |
| ENSRNOG00000000129 | 1.468016 | 5.022943 | -1.77467 | 5.23E-06 | 7.22E-05 | down | Phf24 |
| ENSRNOG00000027606 | 1.965449 | 6.731788 | -1.77613 | 1.71E-06 | 2.58E-05 | down | Neurl1b |
| ENSRNOG00000004921 | 2.713135 | 9.459445 | -1.80179 | 1.58E-09 | 3.99E-08 | down | Nusap1 |
| ENSRNOG00000012278 | 11.72672 | 41.21764 | -1.81346 | 1.86E-24 | 1.78E-22 | down | Fgf10 |
| ENSRNOG00000033772 | 25.97772 | 91.57004 | -1.8176 | 1.13E-41 | 2.91E-39 | down | Serpinb9 |
| ENSRNOG00000005376 | 4.349137 | 15.33249 | -1.81779 | 2.83E-10 | 7.93E-09 | down | Mad2l1 |
| ENSRNOG00000049906 | 4.648311 | 16.46816 | -1.8249 | 5.93E-19 | 3.92E-17 | down | Foxf1 |
| ENSRNOG00000021669 | 3.547965 | 12.61449 | -1.83002 | 3.85E-25 | 3.74E-23 | down | Mybl1 |
| ENSRNOG00000016412 | 1.432584 | 5.095482 | -1.8306 | 3.93E-06 | 5.64E-05 | down | Fxyd6 |
| ENSRNOG00000000818 | 3.068592 | 10.93352 | -1.83311 | 1.39E-08 | 3.05E-07 | down | Nrm |
| ENSRNOG00000002667 | 1.551337 | 5.540356 | -1.83647 | 2.66E-05 | 0.000323 | down | Lamc2 |
| ENSRNOG00000013167 | 8.661237 | 31.07047 | -1.8429 | 3.40E-17 | 1.90E-15 | down | Hmgb2l1 |
| ENSRNOG00000008837 | 1.902698 | 6.878213 | -1.85399 | 4.01E-06 | 5.74E-05 | down | Ass1 |
| ENSRNOG00000039284 | 6.558799 | 23.88463 | -1.86458 | 4.55E-16 | 2.32E-14 | down | Haus4 |
| ENSRNOG00000005700 | 6.012039 | 22.01792 | -1.87275 | 6.62E-16 | 3.35E-14 | down | Nsg1 |
| ENSRNOG00000007118 | 2.165527 | 7.936374 | -1.87376 | 4.72E-09 | 1.12E-07 | down | Eva1a |
| ENSRNOG00000019141 | 1.649077 | 6.081031 | -1.88266 | 1.46E-05 | 0.000186 | down | Ch25h |
| ENSRNOG00000016301 | 4.77398 | 17.70859 | -1.89118 | 4.81E-17 | 2.65E-15 | down | Dmrt2 |
| ENSRNOG00000032929 | 4.478246 | 16.64389 | -1.89399 | 1.09E-22 | 9.35E-21 | down | Incenp |
| ENSRNOG00000013521 | 2.310684 | 8.640102 | -1.90273 | 3.62E-17 | 2.01E-15 | down | Dhfr |
| ENSRNOG00000005185 | 3.067308 | 11.48629 | -1.90487 | 1.34E-11 | 4.36E-10 | down | Nxph3 |
| ENSRNOG00000004972 | 1.85816 | 6.97059 | -1.90741 | 1.23E-08 | 2.74E-07 | down | Upp1 |
| ENSRNOG00000024077 | 2.345263 | 8.84737 | -1.9155 | 1.25E-09 | 3.18E-08 | down | Fbxo5 |
| ENSRNOG00000011648 | 610.6938 | 2311.902 | -1.92056 | 1.56E-64 | 1.27E-61 | down | Aqp1 |
| ENSRNOG00000014130 | 19.94901 | 75.54549 | -1.92103 | 7.99E-23 | 6.98E-21 | down | Cks2 |
| ENSRNOG00000007292 | 1.323641 | 5.032225 | -1.92668 | 8.13E-08 | 1.57E-06 | down | Spdl1 |
| ENSRNOG00000000777 | 3.038444 | 11.6296 | -1.9364 | 5.66E-11 | 1.73E-09 | down | RT1-S3 |
| ENSRNOG00000016257 | 1.774615 | 6.797223 | -1.93744 | 3.27E-06 | 4.74E-05 | down | Cotl1 |
| ENSRNOG00000061085 | 1.390124 | 5.345718 | -1.94317 | 2.58E-11 | 8.13E-10 | down | Fancd2 |
| ENSRNOG00000003908 | 1.791338 | 6.904919 | -1.94659 | 1.21E-06 | 1.89E-05 | down | Cep128 |
| ENSRNOG00000005964 | 1.480037 | 5.748411 | -1.95753 | 5.99E-14 | 2.49E-12 | down | Nr4a3 |
| ENSRNOG00000032626 | 62.03959 | 244.0772 | -1.97608 | 5.02E-06 | 6.98E-05 | down | Mmp3 |
| ENSRNOG00000005291 | 8.52784 | 33.65498 | -1.98057 | 6.82E-28 | 8.64E-26 | down | Slc38a1 |
| ENSRNOG00000014613 | 23.32427 | 92.64815 | -1.98993 | 8.00E-54 | 3.99E-51 | down | Ddah1 |
| ENSRNOG00000010076 | 5.962335 | 23.78198 | -1.99592 | 1.59E-37 | 3.42E-35 | down | Pkp1 |
| ENSRNOG00000020009 | 1.940044 | 7.739038 | -1.99606 | 1.13E-09 | 2.90E-08 | down | Npas4 |
| ENSRNOG00000023410 | 2.452767 | 9.794798 | -1.99761 | 3.97E-09 | 9.58E-08 | down | Apol9a |
| ENSRNOG00000012543 | 4.621746 | 18.56701 | -2.00623 | 3.93E-27 | 4.61E-25 | down | Mcm3 |
| ENSRNOG00000017850 | 4.900368 | 19.80869 | -2.01517 | 7.74E-08 | 1.50E-06 | down | Dctpp1 |
| ENSRNOG00000024650 | 9.162475 | 37.56983 | -2.03577 | 9.22E-34 | 1.60E-31 | down | Ckap2 |
| ENSRNOG00000014372 | 1.292106 | 5.310698 | -2.03918 | 0.000431 | 0.003928 | down | Gjb3 |
| ENSRNOG00000017767 | 6.559272 | 26.97592 | -2.04006 | 1.32E-34 | 2.40E-32 | down | Mrvi1 |
| ENSRNOG00000000488 | 26.65898 | 111.0431 | -2.05843 | 3.36E-14 | 1.43E-12 | down | Hmga1 |
| ENSRNOG00000037199 | 6.263093 | 26.27336 | -2.06865 | 5.87E-12 | 2.00E-10 | down | Spink8 |
| ENSRNOG00000002322 | 4.303427 | 18.07563 | -2.07049 | 2.42E-27 | 2.90E-25 | down | RGD1310587 |
| ENSRNOG00000023720 | 4.266326 | 17.98628 | -2.07583 | 1.99E-29 | 2.75E-27 | down | Ntm |
| ENSRNOG00000020792 | 3.473168 | 14.71301 | -2.08277 | 2.90E-15 | 1.38E-13 | down | Etv4 |
| ENSRNOG00000032832 | 5.994293 | 25.50163 | -2.08893 | 2.06E-14 | 9.06E-13 | down | Mmp10 |
| ENSRNOG00000014080 | 4.337473 | 18.52623 | -2.09464 | 4.21E-20 | 3.07E-18 | down | Kif23 |
| ENSRNOG00000005936 | 3.560791 | 15.40986 | -2.11358 | 1.07E-23 | 9.80E-22 | down | Foxm1 |
| ENSRNOG00000015075 | 4.173023 | 18.47404 | -2.14633 | 1.02E-20 | 7.68E-19 | down | Stc1 |
| ENSRNOG00000015810 | 1.36266 | 6.035394 | -2.14702 | 6.56E-11 | 1.98E-09 | down | Trip13 |
| ENSRNOG00000001692 | 1.297111 | 5.820486 | -2.16584 | 1.56E-07 | 2.84E-06 | down | Chaf1b |
| ENSRNOG00000020875 | 1.365741 | 6.134283 | -2.16721 | 3.87E-07 | 6.57E-06 | down | Celf3 |
| ENSRNOG00000009734 | 6.560826 | 29.61764 | -2.17451 | 8.75E-18 | 5.32E-16 | down | Akr1b10 |
| ENSRNOG00000003703 | 5.19505 | 23.64473 | -2.18631 | 5.19E-35 | 9.68E-33 | down | Mcm6 |
| ENSRNOG00000012879 | 107.8261 | 494.1517 | -2.19625 | 1.14E-30 | 1.63E-28 | down | Fabp3 |
| ENSRNOG00000011296 | 2.66231 | 12.52435 | -2.23399 | 8.02E-17 | 4.29E-15 | down | Cenpn |
| ENSRNOG00000021847 | 1.994163 | 9.645307 | -2.27404 | 6.94E-16 | 3.49E-14 | down | Ska3 |
| ENSRNOG00000027380 | 3.991677 | 19.31302 | -2.27451 | 1.04E-20 | 7.83E-19 | down | Upk1b |
| ENSRNOG00000032778 | 1.378824 | 6.770065 | -2.29573 | 1.17E-15 | 5.67E-14 | down | Bub1 |
| ENSRNOG00000051258 | 9.962073 | 51.49664 | -2.36996 | 3.10E-29 | 4.17E-27 | down | Taf9 |
| ENSRNOG00000002525 | 11.78481 | 61.075 | -2.37365 | 0.001591 | 0.012224 | down | Ptgs2 |
| ENSRNOG00000042286 | 1.055536 | 5.476269 | -2.37522 | 2.90E-09 | 7.15E-08 | down | Nsl1 |
| ENSRNOG00000009334 | 4.380444 | 22.73218 | -2.37559 | 1.67E-25 | 1.66E-23 | down | Knstrn |
| ENSRNOG00000016689 | 1.368585 | 7.157343 | -2.38674 | 1.50E-22 | 1.26E-20 | down | Fanci |
| ENSRNOG00000019174 | 1.007234 | 5.268906 | -2.3871 | 8.00E-14 | 3.30E-12 | down | Chtf18 |
| ENSRNOG00000003657 | 1.320998 | 6.989366 | -2.40353 | 7.59E-12 | 2.54E-10 | down | Pkmyt1 |
| ENSRNOG00000014061 | 1.695328 | 8.971567 | -2.4038 | 2.23E-18 | 1.40E-16 | down | Dusp5 |
| ENSRNOG00000047314 | 8.291637 | 44.28215 | -2.417 | 2.66E-52 | 1.17E-49 | down | Tk1 |
| ENSRNOG00000031431 | 6.100359 | 33.08547 | -2.43923 | 7.49E-34 | 1.32E-31 | down | Cdca8 |
| ENSRNOG00000004479 | 2.783185 | 15.2031 | -2.44956 | 7.95E-27 | 8.78E-25 | down | Aurka |
| ENSRNOG00000018874 | 1.075402 | 5.908834 | -2.458 | 3.13E-17 | 1.76E-15 | down | Phf19 |
| ENSRNOG00000004276 | 8.724245 | 48.07712 | -2.46225 | 9.55E-08 | 1.82E-06 | down | Itga3 |
| ENSRNOG00000008057 | 3.579528 | 19.81065 | -2.46843 | 9.90E-26 | 1.01E-23 | down | Krt7 |
| ENSRNOG00000021713 | 1.676435 | 9.369659 | -2.4826 | 6.28E-28 | 8.03E-26 | down | Kif18b |
| ENSRNOG00000005659 | 2.244746 | 12.57736 | -2.48621 | 9.09E-24 | 8.36E-22 | down | Aurkb |
| ENSRNOG00000023628 | 1.373323 | 7.696619 | -2.48655 | 6.49E-12 | 2.20E-10 | down | Tmem106a |
| ENSRNOG00000003596 | 1.731616 | 9.747418 | -2.4929 | 1.48E-11 | 4.82E-10 | down | Itgb1bp2 |
| ENSRNOG00000042980 | 21.06168 | 118.7495 | -2.49523 | 5.77E-86 | 9.91E-83 | down | Adam19 |
| ENSRNOG00000013727 | 1.628769 | 9.194759 | -2.49703 | 2.98E-19 | 2.02E-17 | down | Ndc80 |
| ENSRNOG00000059894 | 2.276719 | 12.88385 | -2.50054 | 2.16E-15 | 1.04E-13 | down | Hmmr |
| ENSRNOG00000003927 | 6.551141 | 37.29281 | -2.50908 | 1.17E-40 | 2.92E-38 | down | Cd55 |
| ENSRNOG00000046635 | 0.993055 | 5.692481 | -2.51911 | 1.80E-07 | 3.24E-06 | down | LOC100910252 |
| ENSRNOG00000018782 | 2.109537 | 12.10289 | -2.52035 | 1.25E-13 | 5.10E-12 | down | Gmnn |
| ENSRNOG00000055111 | 3.983465 | 23.1049 | -2.5361 | 3.72E-26 | 3.97E-24 | down | AABR07000658.1 |
| ENSRNOG00000016810 | 33.40442 | 195.0383 | -2.54565 | 1.09E-74 | 1.13E-71 | down | Stmn1 |
| ENSRNOG00000006731 | 2.311782 | 13.59622 | -2.55613 | 6.74E-12 | 2.27E-10 | down | Spc25 |
| ENSRNOG00000005465 | 8.400225 | 49.65764 | -2.56352 | 0.001213 | 0.009704 | down | Kcnmb1 |
| ENSRNOG00000043342 | 12.35904 | 73.10828 | -2.56447 | 1.11E-15 | 5.41E-14 | down | LOC290595 |
| ENSRNOG00000015465 | 0.956201 | 5.665825 | -2.5669 | 7.02E-12 | 2.36E-10 | down | Cep72 |
| ENSRNOG00000002711 | 2.379281 | 14.1936 | -2.57664 | 1.56E-17 | 9.05E-16 | down | Nuf2 |
| ENSRNOG00000012051 | 2.231721 | 13.34111 | -2.57965 | 1.18E-30 | 1.67E-28 | down | Ncaph |
| ENSRNOG00000027024 | 33.2229 | 200.1405 | -2.59076 | 1.44E-05 | 0.000183 | down | Rgs16 |
| ENSRNOG00000024733 | 1.030704 | 6.29817 | -2.6113 | 3.78E-05 | 0.000442 | down | Mnd1 |
| ENSRNOG00000009785 | 11.83497 | 73.96626 | -2.64381 | 7.49E-47 | 2.36E-44 | down | Cdkn3 |
| ENSRNOG00000019587 | 7.33156 | 47.11354 | -2.68395 | 3.77E-57 | 2.16E-54 | down | Ptprn |
| ENSRNOG00000053047 | 2.774973 | 17.94753 | -2.69324 | 5.50E-28 | 7.10E-26 | down | Top2a |
| ENSRNOG00000002843 | 4.905436 | 31.75301 | -2.69444 | 0.000208 | 0.002071 | down | Cxcl6 |
| ENSRNOG00000022911 | 1.00518 | 6.538273 | -2.70146 | 6.05E-18 | 3.74E-16 | down | Hjurp |
| ENSRNOG00000048411 | 1.639369 | 10.6939 | -2.70558 | 2.20E-34 | 3.90E-32 | down | Uhrf1 |
| ENSRNOG00000053026 | 0.947147 | 6.196939 | -2.7099 | 2.06E-07 | 3.67E-06 | down | Shcbp1 |
| ENSRNOG00000008040 | 4.366702 | 28.8786 | -2.72538 | 1.01E-25 | 1.02E-23 | down | Fam64a |
| ENSRNOG00000025302 | 0.93963 | 6.217577 | -2.72619 | 6.09E-18 | 3.75E-16 | down | Cdca2 |
| ENSRNOG00000029401 | 26.46156 | 177.3108 | -2.74431 | 2.26E-08 | 4.83E-07 | down | Actg2 |
| ENSRNOG00000020281 | 4.985099 | 33.49342 | -2.74818 | 6.33E-53 | 2.97E-50 | down | Kif22 |
| ENSRNOG00000017484 | 5.547103 | 37.27251 | -2.74831 | 1.68E-31 | 2.54E-29 | down | Gja5 |
| ENSRNOG00000024365 | 1.55339 | 10.45871 | -2.75121 | 6.99E-16 | 3.50E-14 | down | Ect2 |
| ENSRNOG00000003229 | 1.555311 | 10.51155 | -2.7567 | 3.29E-17 | 1.85E-15 | down | Tspan7 |
| ENSRNOG00000024428 | 8.286659 | 56.5998 | -2.77193 | 5.10E-54 | 2.63E-51 | down | Kif20a |
| ENSRNOG00000015423 | 5.113246 | 35.27135 | -2.78619 | 6.80E-31 | 9.82E-29 | down | Ccna2 |
| ENSRNOG00000003802 | 3.065188 | 21.17275 | -2.78816 | 1.04E-19 | 7.38E-18 | down | Pttg1 |
| ENSRNOG00000013598 | 1.062117 | 7.337029 | -2.78825 | 1.28E-12 | 4.63E-11 | down | Melk |
| ENSRNOG00000000632 | 17.40554 | 120.6696 | -2.79344 | 3.76E-58 | 2.33E-55 | down | Cdk1 |
| ENSRNOG00000015308 | 1.89491 | 13.25434 | -2.80626 | 7.88E-24 | 7.29E-22 | down | Pbk |
| ENSRNOG00000060703 | 1.798852 | 12.59467 | -2.80767 | 5.45E-23 | 4.81E-21 | down | Troap |
| ENSRNOG00000013057 | 7.294335 | 51.21326 | -2.81167 | 6.02E-52 | 2.59E-49 | down | Prc1 |
| ENSRNOG00000004487 | 1.884618 | 13.23554 | -2.81207 | 9.36E-19 | 6.11E-17 | down | Nek2 |
| ENSRNOG00000023262 | 1.596417 | 11.21655 | -2.81272 | 1.03E-16 | 5.48E-15 | down | Cenpm |
| ENSRNOG00000057153 | 5.059301 | 35.71426 | -2.81949 | 8.74E-45 | 2.50E-42 | down | Pla1a |
| ENSRNOG00000018735 | 0.816174 | 5.769377 | -2.82147 | 7.49E-05 | 0.000823 | down | Cd74 |
| ENSRNOG00000008986 | 4.71395 | 33.54419 | -2.83105 | 6.30E-48 | 2.07E-45 | down | Diaph3 |
| ENSRNOG00000049033 | 3.591984 | 25.74502 | -2.84144 | 3.57E-36 | 7.16E-34 | down | Racgap1 |
| ENSRNOG00000005871 | 5.724727 | 41.19305 | -2.84712 | 1.40E-47 | 4.53E-45 | down | Il1rn |
| ENSRNOG00000000479 | 3.584658 | 25.79893 | -2.8474 | 1.23E-46 | 3.80E-44 | down | Kifc1 |
| ENSRNOG00000016377 | 1.143154 | 8.271169 | -2.85507 | 3.84E-19 | 2.58E-17 | down | Cep55 |
| ENSRNOG00000015529 | 4.568214 | 33.66221 | -2.88143 | 1.26E-38 | 2.78E-36 | down | Cdca3 |
| ENSRNOG00000008115 | 1.357997 | 10.10228 | -2.89513 | 8.51E-32 | 1.33E-29 | down | Arhgap11a |
| ENSRNOG00000042944 | 1.48965 | 11.105 | -2.89816 | 5.38E-20 | 3.89E-18 | down | Cenpw |
| ENSRNOG00000013970 | 0.990877 | 7.393376 | -2.89946 | 1.11E-16 | 5.84E-15 | down | Cdt1 |
| ENSRNOG00000014960 | 0.868447 | 6.506004 | -2.90526 | 1.08E-21 | 8.51E-20 | down | Bard1 |
| ENSRNOG00000007906 | 1.467961 | 11.0337 | -2.91003 | 4.44E-39 | 1.06E-36 | down | Bub1b |
| ENSRNOG00000026143 | 1.43655 | 10.84995 | -2.91701 | 2.49E-27 | 2.96E-25 | down | Ckap2l |
| ENSRNOG00000015794 | 1.630484 | 12.44387 | -2.93206 | 2.14E-28 | 2.83E-26 | down | Fam83d |
| ENSRNOG00000010797 | 9.896638 | 75.89639 | -2.93902 | 1.07E-86 | 2.07E-83 | down | Esm1 |
| ENSRNOG00000015275 | 0.878073 | 6.762436 | -2.94513 | 1.31E-08 | 2.88E-07 | down | Ska1 |
| ENSRNOG00000023465 | 3.772386 | 29.07383 | -2.94617 | 0.000111 | 0.001169 | down | LOC500300 |
| ENSRNOG00000004649 | 1.784629 | 13.79514 | -2.95046 | 0.006338 | 0.038478 | down | Il1b |
| ENSRNOG00000019100 | 1.995059 | 15.474 | -2.95534 | 1.48E-41 | 3.76E-39 | down | Kif2c |
| ENSRNOG00000018355 | 0.817979 | 6.353965 | -2.95752 | 4.35E-14 | 1.84E-12 | down | Msx2 |
| ENSRNOG00000045924 | 0.737204 | 5.729728 | -2.95833 | 3.12E-08 | 6.44E-07 | down | RT1-T24-3 |
| ENSRNOG00000006033 | 28.10232 | 219.4055 | -2.96484 | 1.56E-12 | 5.55E-11 | down | Spon2 |
| ENSRNOG00000028865 | 1.435355 | 11.23608 | -2.96866 | 3.52E-32 | 5.73E-30 | down | Kprp |
| ENSRNOG00000017259 | 4.27516 | 33.77545 | -2.98192 | 5.49E-40 | 1.35E-37 | down | Tacc3 |
| ENSRNOG00000018615 | 0.689861 | 5.492995 | -2.99322 | 7.77E-09 | 1.79E-07 | down | Cenph |
| ENSRNOG00000023633 | 1.03619 | 8.34462 | -3.00956 | 7.21E-07 | 1.17E-05 | down | Crabp1 |
| ENSRNOG00000029862 | 5.603348 | 45.2681 | -3.01413 | 8.51E-39 | 1.96E-36 | down | Spc24 |
| ENSRNOG00000008165 | 3.465177 | 28.72841 | -3.05148 | 5.25E-48 | 1.77E-45 | down | Tpx2 |
| ENSRNOG00000014336 | 2.906646 | 24.42202 | -3.07076 | 3.77E-50 | 1.42E-47 | down | Mcm5 |
| ENSRNOG00000013774 | 2.603292 | 22.0615 | -3.08312 | 1.58E-45 | 4.71E-43 | down | Lmnb1 |
| ENSRNOG00000056069 | 0.87941 | 7.474551 | -3.08738 | 4.97E-05 | 0.000568 | down | Kif11 |
| ENSRNOG00000028415 | 7.537326 | 64.22989 | -3.09112 | 2.26E-52 | 1.03E-49 | down | Cdc20 |
| ENSRNOG00000024178 | 0.8872 | 7.603645 | -3.09936 | 1.89E-18 | 1.20E-16 | down | Cenpt |
| ENSRNOG00000011777 | 1.582774 | 13.58789 | -3.10179 | 1.84E-48 | 6.33E-46 | down | Spag5 |
| ENSRNOG00000014343 | 5.93125 | 51.23324 | -3.11067 | 2.96E-87 | 6.53E-84 | down | Anln |
| ENSRNOG00000021555 | 0.641544 | 5.54231 | -3.11087 | 4.00E-11 | 1.24E-09 | down | Mis18a |
| ENSRNOG00000038035 | 0.723762 | 6.303491 | -3.12256 | 1.51E-23 | 1.36E-21 | down | Kif4a |
| ENSRNOG00000061376 | 2.558672 | 22.47058 | -3.13457 | 4.62E-10 | 1.25E-08 | down | Psca |
| ENSRNOG00000050819 | 4.127146 | 36.39603 | -3.14056 | 2.59E-30 | 3.64E-28 | down | Birc5 |
| ENSRNOG00000046246 | 19.97505 | 177.3508 | -3.15033 | 1.62E-51 | 6.76E-49 | down | Spon2 |
| ENSRNOG00000042460 | 0.830417 | 7.389657 | -3.1536 | 1.12E-08 | 2.52E-07 | down | Hmga2 |
| ENSRNOG00000010997 | 0.701351 | 6.307565 | -3.16887 | 1.31E-15 | 6.34E-14 | down | Ednrb |
| ENSRNOG00000032596 | 1.911433 | 17.23166 | -3.17234 | 1.01E-21 | 7.94E-20 | down | RT1-T24-1 |
| ENSRNOG00000018113 | 1.265956 | 11.47911 | -3.18071 | 7.65E-37 | 1.60E-34 | down | Anlnl1 |
| ENSRNOG00000058539 | 4.903588 | 44.86282 | -3.19361 | 3.61E-64 | 2.79E-61 | down | Ccnb1 |
| ENSRNOG00000028137 | 1.216844 | 11.32545 | -3.21835 | 1.31E-45 | 3.96E-43 | down | Mki67 |
| ENSRNOG00000016561 | 7.20839 | 67.11394 | -3.21886 | 3.06E-22 | 2.49E-20 | down | Ns5atp9 |
| ENSRNOG00000005038 | 2.078275 | 19.54185 | -3.23311 | 2.08E-16 | 1.09E-14 | down | Ube2t |
| ENSRNOG00000037302 | 0.810422 | 7.637419 | -3.23634 | 8.83E-19 | 5.79E-17 | down | Rad51 |
| ENSRNOG00000027787 | 0.57908 | 5.501059 | -3.24788 | 6.13E-20 | 4.39E-18 | down | Cdc6 |
| ENSRNOG00000006198 | 0.547461 | 5.250912 | -3.26174 | 3.41E-14 | 1.44E-12 | down | Prr11 |
| ENSRNOG00000015131 | 9.599811 | 94.14738 | -3.29384 | 1.50E-56 | 8.26E-54 | down | Ube2c |
| ENSRNOG00000054286 | 9.865743 | 97.269 | -3.30148 | 4.82E-71 | 4.14E-68 | down | Rrm2 |
| ENSRNOG00000008450 | 5.245318 | 51.88576 | -3.30624 | 1.46E-53 | 7.05E-51 | down | LOC100359539 |
| ENSRNOG00000032178 | 1.121744 | 11.10708 | -3.30766 | 1.64E-31 | 2.51E-29 | down | Cenpa |
| ENSRNOG00000018815 | 3.78555 | 37.61176 | -3.31261 | 1.25E-79 | 1.49E-76 | down | Plk1 |
| ENSRNOG00000008091 | 1.152195 | 11.56171 | -3.3269 | 1.33E-17 | 7.74E-16 | down | Gins1 |
| ENSRNOG00000009339 | 0.554467 | 5.646792 | -3.34826 | 4.79E-35 | 9.04E-33 | down | Cenpe |
| ENSRNOG00000000036 | 0.969937 | 9.932221 | -3.35615 | 2.95E-28 | 3.86E-26 | down | Klhdc8a |
| ENSRNOG00000057092 | 0.858737 | 8.959479 | -3.38313 | 2.86E-32 | 4.71E-30 | down | Slfn4 |
| ENSRNOG00000025198 | 0.65173 | 6.94044 | -3.41268 | 1.64E-34 | 2.94E-32 | down | Gas2l3 |
| ENSRNOG00000027894 | 1.257256 | 13.68597 | -3.44435 | 1.14E-71 | 1.03E-68 | down | Iqgap3 |
| ENSRNOG00000046301 | 0.730839 | 7.955969 | -3.44441 | 1.22E-09 | 3.11E-08 | down | Gzmbl3 |
| ENSRNOG00000019549 | 20.69935 | 225.7696 | -3.44719 | ######## | ######## | down | Akap12 |
| ENSRNOG00000007805 | 1.031957 | 11.31329 | -3.45456 | 6.99E-43 | 1.90E-40 | down | Mybl2 |
| ENSRNOG00000007483 | 0.991152 | 11.34704 | -3.51707 | 2.75E-43 | 7.59E-41 | down | Ccnf |
| ENSRNOG00000004575 | 1.095164 | 13.1715 | -3.5882 | 2.52E-13 | 9.97E-12 | down | Il1a |
| ENSRNOG00000005115 | 1.733376 | 22.26197 | -3.68292 | 1.32E-35 | 2.55E-33 | down | Asf1b |
| ENSRNOG00000010906 | 2.553778 | 38.97928 | -3.932 | 0.000421 | 0.003856 | down | Ccl5 |
| ENSRNOG00000009768 | 2.399677 | 39.13124 | -4.02741 | 3.12E-05 | 0.000371 | down | Npy |
| ENSRNOG00000013069 | 0.797851 | 13.18326 | -4.04645 | 1.42E-31 | 2.20E-29 | down | Sapcd2 |
| ENSRNOG00000046449 | 3.072282 | 53.45536 | -4.12095 | 1.13E-06 | 1.77E-05 | down | LOC100912228 |
| ENSRNOG00000048967 | 3.703949 | 67.02196 | -4.1775 | 9.08E-99 | 3.51E-95 | down | LOC688459 |
| ENSRNOG00000057443 | 0.999468 | 19.48511 | -4.28507 | 0.005751 | 0.035655 | down | LOC497963 |
| ENSRNOG00000018606 | 0.344171 | 6.718529 | -4.28695 | 5.15E-32 | 8.26E-30 | down | Olr59 |
| ENSRNOG00000042105 | 0.974894 | 27.08977 | -4.79636 | 5.18E-32 | 8.26E-30 | down | Lce1l |
| ENSRNOG00000058370 | 0.232197 | 6.751421 | -4.86177 | 1.34E-07 | 2.49E-06 | down | AABR07053481.1 |
| ENSRNOG00000030111 | 0.197808 | 6.260254 | -4.98405 | 2.19E-22 | 1.80E-20 | down | Cyp11b2 |
| ENSRNOG00000058340 | 0.331659 | 13.08273 | -5.30182 | 1.50E-42 | 4.01E-40 | down | Krt79 |
| ENSRNOG00000009581 | 0.187133 | 8.144517 | -5.4437 | 1.28E-13 | 5.20E-12 | down | Lce1m |
| ENSRNOG00000053766 | 0.53324 | 32.15104 | -5.91394 | 9.37E-59 | 6.04E-56 | down | Ramp3 |
| ENSRNOG00000050963 | 0.280207 | 21.86884 | -6.28624 | 0.00307 | 0.021052 | down | AABR07064716.3 |
| ENSRNOG00000016535 | 0.343882 | 53.84465 | -7.29075 | 5.05E-24 | 4.70E-22 | down | Ccl22 |
